# Stage Migration, Treatment Patterns, and Machine Learning-Based Prediction of 12-Month Mortality in Lung Adenocarcinoma Across the COVID-19 Era: A SEER Population-Based Study

**DOI:** 10.3390/diagnostics16203369

**Published:** 2026-10-16

**Authors:** Ömer Emin Çınar, Yasemin Adalı

**Affiliations:** 1 School of Electronics, Electrical Engineering and Computer Science, Queen’s University Belfast, Belfast BT9 5BN, UK; 2 Department of Computer Engineering, Faculty of Engineering and Architecture, Recep Tayyip Erdogan University, Rize 53100, Türkiye; 3 Centre for Public Health, School of Medicine, Dentistry and Biomedical Sciences, Queen’s University Belfast, Belfast BT7 1NN, UK; y.adali@qub.ac.uk; 4 Division of Epidemiology, Department of Public Health, Faculty of Medicine, Pamukkale University, Denizli 20070, Türkiye

**Keywords:** lung adenocarcinoma, COVID-19, SEER, clinical prediction model, calibration, decision curve analysis, machine learning, epidemiology

## Abstract

**Background/Objectives**: The COVID-19 pandemic coincided with widespread disruption of cancer care. We examined changes in lung adenocarcinoma presentation and outcomes across the COVID-19 era and whether diagnosis-time information identifies patients at risk of early death. **Methods**: Adults with microscopically confirmed lung adenocarcinoma diagnosed during 2018–2023 were identified from the Surveillance, Epidemiology, and End Results 17 Registries database and grouped into three diagnostic periods. Logistic regression, time-varying Cox, and Fine-Gray models evaluated stage, metastasis, surgery, delay, and mortality. Four machine-learning algorithms using only diagnosis-time variables were developed (2018–2021; *n* = 87,281) and temporally validated (2022; *n* = 22,012). **Results**: Among 133,306 patients, COVID-19 versus pre-COVID-19 differences were significant but small in absolute terms: stage IV disease 47.9% versus 47.4% (adjusted odds ratio [OR], 1.03; 95% CI, 1.00–1.06), organ-specific metastasis 45.8% versus 44.4% (OR, 1.06; 1.04–1.09), and treatment delay exceeding 90 days 11.1% versus 10.2% (OR, 1.11; 1.06–1.17). Post-COVID-19 (2022–2023) diagnosis showed less advanced disease but substantially longer delay (14.5% versus 10.2%; OR, 1.45; 1.39–1.52). Gradient boosting achieved a 2022 ROC AUC of 0.840 (0.834–0.845); it flagged 46.8% of patients and correctly flagged 81.6% of those who died, against 44.0% and 75.4% for stage IV classification. **Conclusions**: COVID-era changes in stage and metastatic presentation were small in absolute terms, whereas the increase in treatment delay was larger and persisted. A diagnosis-time model modestly improved identification of early death beyond stage alone, flagging 28 more patients per 1000, of whom 21 died within the year. These findings support further evaluation; external validation is required before clinical use.

## 1. Introduction

Lung cancer remains the most frequently diagnosed malignancy and the leading cause of cancer-related death worldwide, accounting for approximately 2.5 million new cases and 1.8 million deaths in 2022 [1]. Adenocarcinoma is the predominant histological subtype, representing an estimated 45.6% of lung cancers among men and 59.7% among women globally [2]. Despite advances in screening, molecular characterisation, and systemic treatment, prognosis remains strongly dependent on disease extent at diagnosis. Consequently, disruptions affecting early detection, diagnostic work-up, or timely treatment may have substantial implications for both treatment eligibility and survival.

The COVID-19 pandemic caused widespread interruptions across cancer-care pathways through temporary suspension of screening programs, reduced diagnostic activity, constrained healthcare capacity, and changes in treatment delivery. Population-based data from the United States showed that lung cancer incidence declined by 13.6% between 2019 and 2020, accompanied by an increased proportion of patients diagnosed with distant-stage disease [3]. Analyses of patients with non-small-cell lung cancer similarly reported more advanced presentation, lower treatment receipt, and reduced use of primary surgical resection during the pandemic [4]. However, the observed effects have not been uniform. Some institutional studies identified increases in stage IV disease or longer surgical waiting times [5], whereas others reported preservation of treatment delivery and short-term survival despite diagnostic disruptions [6]. These inconsistencies may reflect differences in population coverage, pandemic-period definitions, healthcare-system resilience, histological composition, and the outcomes examined.

Moreover, evidence regarding recovery after the acute pandemic period remains limited. Recent national findings suggest that stage distributions may have partially improved after the initial disruption, while time to treatment and changes in surgical practice may have persisted [7]. Most previous studies have focused on lung cancer or non-small-cell lung cancer as broad clinical entities and have compared only pre-pandemic and pandemic periods. Fewer studies have specifically examined lung adenocarcinoma across the pre-COVID-19, COVID-19, and post-COVID-19 periods while simultaneously evaluating the stage at diagnosis, organ-specific metastatic involvement, pulmonary resection, type of resection, treatment delay, and short-term mortality. Distinguishing a transient pandemic-associated disruption from a persistent post-pandemic change requires population-based follow-up extending beyond 2021.

Period-level comparisons of this kind describe how a population changed, but they do not help a clinician meeting an individual patient. If treatment delays persist after the acute pandemic period, clinicians must decide which patients to prioritise for earlier supportive-care involvement, expedited multidisciplinary review, or more intensive follow-up, and that decision is made at the moment of diagnosis on the basis of that patient’s own characteristics. In a randomised trial of patients with newly diagnosed metastatic non-small-cell lung cancer, early integration of specialist palliative care improved quality of life and mood and reduced aggressive end-of-life care [8], and the principal practical obstacle to delivering it is identifying candidates promptly rather than late in the disease course. Individual-level risk estimation is therefore a distinct and complementary task to population-level epidemiology, and it is the task to which prediction modelling is suited. Machine-learning methods may complement conventional epidemiological analyses by identifying patients at high risk of early mortality. Nevertheless, a clinically meaningful prognostic model should use predictors available at its intended time of application and should be evaluated in patients who are temporally distinct from those used for model development. Inclusion of treatment variables or events occurring after diagnosis can introduce information unavailable at initial presentation and artificially inflate apparent predictive performance. Current TRIPOD + AI (Transparent Reporting of a multivariable prediction model for Individual Prognosis Or Diagnosis extended to artificial intelligence) guidance therefore emphasises explicit definition of the intended use, timing of predictor measurement, rigorous internal validation, temporal or external evaluation, and assessment of both discrimination and calibration [9]. A further requirement, less often addressed, is that the incremental value of a model be judged against the risk classification already in routine use, which, for lung cancer at diagnosis, is stage.

The Surveillance, Epidemiology, and End Results (SEER) Program of the National Cancer Institute is the principal population-based cancer surveillance system in the United States and the only national source that combines stage at diagnosis with long-term follow-up for vital status. Participating registries operate under state public-health reporting authority and must ascertain all incident cancers among residents of their catchment areas, rather than only those treated at academic or referral centres, which substantially reduces the selection bias affecting single-institution and trial-based cohorts. The SEER 17 registry configuration used here covers approximately one quarter of the United States population and was designed to be demographically and geographically representative, with deliberate oversampling of minority populations [10]. Registries routinely abstract patient demographics, primary tumour site, histology, grade, stage, tumour size, organ-specific metastatic involvement, first course of treatment, and date of treatment initiation, and ascertain vital status through active follow-up and linkage with the National Death Index. Data are subject to standardised coding rules, electronic pathology reporting, and ongoing quality-control and registrar-training programs coordinated with the North American Association of Central Cancer Registries. These characteristics make SEER well-suited to examining whether presentation and care changed across the COVID-19 era at the population scale and to developing a prognostic model from variables that are reliably documented at the time of diagnosis in routine practice. Equally relevant to the present study, SEER does not capture performance status, comorbidity burden, smoking history, molecular alterations, or treatment beyond the first course, and these constraints shape both what can be inferred about care pathways and what a prediction model built on these data can be expected to achieve [11].

Accordingly, this population-based study used SEER data to examine stage distribution, organ-specific metastasis, treatment patterns, treatment delay, and 12-month mortality among adults with microscopically confirmed lung adenocarcinoma diagnosed before, during, and after the COVID-19 pandemic. Because changes in treatment timing were found to persist beyond the acute pandemic period, we additionally examined whether patients at high risk of early death can be identified at the moment of diagnosis, when prioritisation decisions are actually made. We therefore developed and temporally validated machine-learning models predicting 12-month all-cause mortality from information available at initial presentation, and evaluated them not only for discrimination but for calibration, clinical utility, and incremental value over stage-based risk classification as it is currently used in practice. Finally, we quantified the apparent performance gain produced by adding post-diagnosis treatment variables, thereby assessing the extent to which temporally unavailable information may overstate model performance.

## 2. Materials and Methods

### 2.1. Study Design and Data Source

This population-based retrospective cohort study used data from the Surveillance, Epidemiology, and End Results (SEER) Program of the National Cancer Institute, drawn from the Incidence-SEER Research Data, 17 Registries, November 2025 Submission (2000–2023) and linked to time-dependent county attributes. The study was designed and reported in accordance with the Strengthening the Reporting of Observational Studies in Epidemiology (STROBE) recommendations [12] and with the TRIPOD + AI statement; completed checklists for both are provided as Supplementary Tables S9 and S10. Because SEER is a population-based cancer registry rather than a clinical record, several variables that strongly influence prognosis and treatment selection in lung adenocarcinoma are not collected. These include Eastern Cooperative Oncology Group performance status, comorbidity burden, smoking history and pack-years, pulmonary function, molecular and biomarker status such as epidermal growth factor receptor (EGFR), anaplastic lymphoma kinase (ALK), ROS proto-oncogene 1 (ROS1), and programmed death-ligand 1 (PD-L1), treatment beyond the first course, and individual severe acute respiratory syndrome coronavirus 2 (SARS-CoV-2) infection status or local pandemic intensity. The consequences of these omissions for the interpretation of both the epidemiological and the prediction analyses are considered in Section 4.

### 2.2. Study Population and Cohort Selection

**Figure 1:**
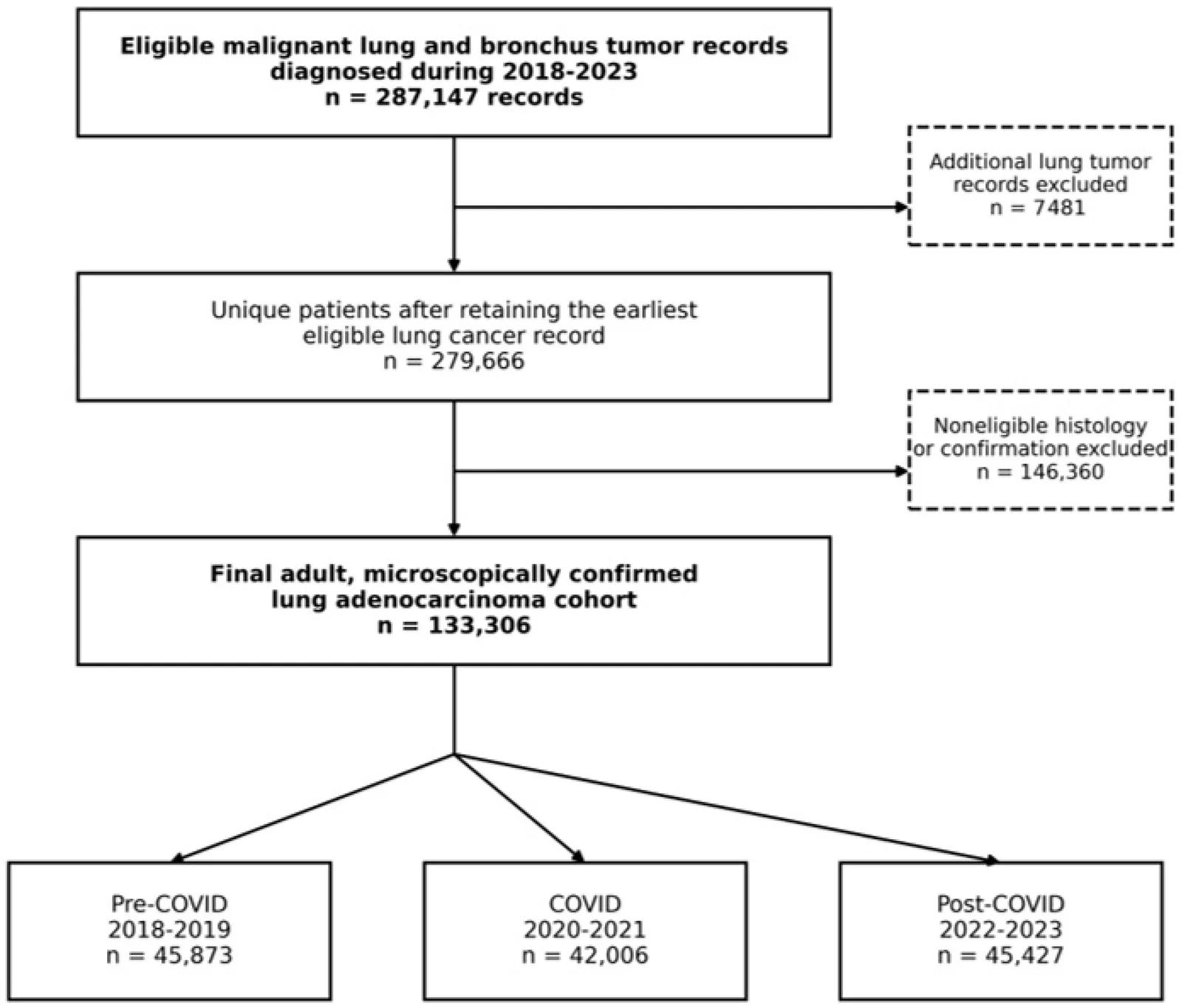
Derivation of the final SEER lung adenocarcinoma study cohort. Solid boxes and arrows indicate the sequence of cohort derivation; dashed boxes indicate records or patients excluded at each stage. Numbers show tumour records or unique patients remaining after each stage and the number removed by the criteria applied at that stage. Autopsy-only and death-certificate-only cases were excluded during the SEER extraction and are not quantified here. The final cohort of 133,306 patients is shown by diagnostic period. Period labels in the lower row denote pre-COVID-19 (2018–2019), COVID-19 (2020–2021), and post-COVID-19 (2022–2023); this three-period categorisation applies to the descriptive and regression analyses.

The initial extraction was restricted to malignant primary tumours classified as “Lung and Bronchus” according to the SEER Site Recode ICD-O-3 (International Classification of Diseases for Oncology, third edition)/WHO 2008, with malignant behaviour according to ICD-O-3, diagnosed between 1 January 2018 and 31 December 2023, in patients aged 18 years or older with an eligible reporting source and sufficient identifiers for patient-level processing. Cases reported only by autopsy and cases reported only by death certificate were excluded during the SEER extraction and were therefore not present in the downloaded records; their number cannot be reconstructed from the analytic dataset and is not included in the exclusion counts reported below. The complete inclusion and exclusion criteria and the number of records or patients removed at each stage are given in Table 1, and the derivation of the cohort is shown in Figure 1.

The merged adult lung cancer dataset contained 287,147 eligible tumour records representing 279,666 unique patients. Patients were ordered according to patient identifier, diagnosis year, and SEER record number. For patients with more than one eligible lung cancer record, only the chronologically earliest eligible record was retained, which removed 7481 additional tumour records and produced a patient-level dataset of 279,666 individuals. The retained record is therefore described as the patient’s earliest eligible lung cancer record within the study dataset; it should not be interpreted as the patient’s first-ever malignant primary tumour because the selection was performed only among the eligible lung cancer records available for this study.

Lung adenocarcinoma was identified using a prespecified expanded definition based on ICD-O-3 histology codes, which included conventional adenocarcinoma and relevant histological variants: acinar, papillary, bronchioloalveolar/lepidic, solid, mucinous, adenosquamous, and other adenocarcinoma subtypes. The complete ICD-O-3 code list is provided in the Supplementary Methods. Inclusion additionally required microscopic confirmation, defined as positive histology, positive exfoliative cytology, positive microscopic confirmation with the method not specified, or positive histology supported by immunophenotyping and/or genetic studies. Cases diagnosed solely by clinical assessment, radiography, laboratory-marker studies, or an unknown method were not considered adequately confirmed and were excluded. Of the 279,666 unique adult patients with an eligible lung cancer record, 146,360 did not meet the combined histological and diagnostic-confirmation criteria. This figure includes 1625 records that met the SEER-defined adenocarcinoma histology but lacked acceptable microscopic confirmation; these are counted once within the combined exclusion and not separately.

The final analytic cohort therefore comprised 133,306 adults with microscopically confirmed lung adenocarcinoma. The full cohort was used for descriptive comparisons of stage, metastasis, and treatment, with missing categories displayed explicitly. Complete-case restrictions were applied only within the corresponding adjusted models, so the analysed sample size varies between models; analysis-specific populations and the restrictions producing them are reported in Supplementary Table S2. Survival analyses were restricted to diagnosis years with sufficient follow-up, and the exclusion of 2023 diagnoses from those analyses is not treated as an exclusion from the main epidemiological cohort.

### 2.3. Definition of the COVID-19 Diagnostic Periods

Patients were categorised into three mutually exclusive groups according to the year of diagnosis: the pre-COVID-19 period (2018–2019), the COVID-19 period (2020–2021), and the post-COVID-19 period (2022–2023). These labels denote calendar periods relative to the acute phase of the pandemic and are used throughout for consistency with the analysis design. They are not intended to indicate that healthcare systems had returned to pre-pandemic functioning: as reported below, treatment delays during 2022–2023 were longer than before the pandemic, so the period designated post-COVID-19 should be understood as the period following the acute phase rather than as a period of restored capacity. The final cohort included 45,873 patients diagnosed before COVID-19, 42,006 diagnosed during COVID-19, and 45,427 diagnosed after COVID-19. Because patients diagnosed in 2023 did not have complete 12-month follow-up, survival analyses were restricted to diagnoses occurring between 2018 and 2022. Accordingly, the survival-period categories were defined as pre-COVID-19 (2018–2019), COVID-19 (2020–2021), and early post-COVID-19 (2022). To avoid ambiguity, the two categorisations are distinguished by name throughout the manuscript. The three-period categorisation used in descriptive and regression analyses is written as post-COVID-19 (2022–2023), and the categorisation used in survival analyses, from which 2023 is necessarily excluded, is written as early post-COVID-19 (2022). The defining year range is stated in the title of every table and figure in which a period label appears. The distribution of the final cohort by diagnostic period is provided in Supplementary Table S1.

### 2.4. Demographic and Socioeconomic Variables

Demographic variables included age at diagnosis, sex, and marital status. Age was analysed as a continuous variable and categorised as 18–49, 50–59, 60–69, 70–79, and ≥80 years for descriptive analyses. Marital status was grouped as married or partnered, single, divorced or separated, widowed, and unknown.

Socioeconomic characteristics included county-level median household income, adjusted to 2024 US dollars, and rural–urban residence. Median household income was categorised as <$70,000, $70,000–$89,999, $90,000–$109,999, and ≥$110,000. Rural–urban residence was derived from the Rural–Urban Continuum Code and categorised as large metropolitan, small or medium metropolitan, and nonmetropolitan. Patient-level race and ethnicity were not included because the corresponding variable in the analytic extraction contained only the aggregate category “All races/ethnicities” and therefore provided no individual-level variation.

### 2.5. Tumour Characteristics

Tumour characteristics included primary tumour site, laterality, derived summary grade, diagnostic confirmation, tumour size, stage at diagnosis, organ-specific metastases, and the presence of separate ipsilateral pulmonary nodules. Primary tumour site was classified as the main bronchus, upper lobe, middle lobe, lower lobe, overlapping lung lesion, or lung not otherwise specified.

Stage at diagnosis was derived from the SEER Derived Extent of Disease (EOD) 2018 Stage Group Recode. Detailed stage categories were consolidated into stages I, II, III, and IV. Codes indicating an unknown, inapplicable, or unassessable stage were classified as unknown. Two binary stage outcomes were subsequently generated: advanced-stage disease, defined as stage III–IV versus stage I–II, and metastatic-stage disease, defined as stage IV versus stage I–III. Patients with unknown stage were excluded from the regression models in which stage was the dependent variable.

Tumour size was obtained from the SEER Tumour Size Summary variable and converted to millimetres. SEER codes indicating an unknown or nonmeasurable tumour size were treated as missing. Tumour size was categorised as ≤20, 21–30, 31–50, 51–70, and >70 mm. Measurements exceeding the prespecified anatomical plausibility limits were also treated as missing. The coding and data-cleaning rules applied to tumour size are detailed in the Supplementary Methods.

Metastatic disease at diagnosis was evaluated using SEER variables for bone, brain, liver, lung, distant lymph node, and other distant metastases. An “any organ-specific metastasis” variable was coded as present when at least one of these metastatic sites was recorded as positive and as absent only when all component variables were recorded as negative. Patients without sufficient information to determine metastatic status were classified as missing. Separate ipsilateral tumour nodules were categorised according to whether nodules were absent, located in the same lobe, located in a different ipsilateral lobe, located in both the same and different lobes, or present in an unspecified ipsilateral lobe.

### 2.6. Treatment Characteristics

Treatment variables included primary-site surgery, type of surgery, pulmonary resection, radiotherapy, chemotherapy, and time from diagnosis to treatment. Surgical procedures were grouped as no surgery, local tumour destruction, sublobar resection, lobectomy, extended lobectomy, pneumonectomy, extended or radical pneumonectomy, and resection or surgery not otherwise specified. Pulmonary resection was defined as a SEER surgery code corresponding to a resection procedure, whereas no surgery and local tumour-destructive procedures without pulmonary resection were classified as no pulmonary resection.

Radiotherapy and chemotherapy were classified using the corresponding SEER treatment variables. Because SEER combines “No” and “Unknown” for these variables, the reference category was reported as “No/unknown” and was not interpreted as definitive evidence that treatment was not administered. Time from diagnosis to treatment was categorised as same day, 1–30 days, 31–60 days, 61–90 days, and >90 days. A prolonged treatment delay was defined as treatment initiation more than 90 days after diagnosis. Patients with unknown treatment timing were excluded from the adjusted treatment-delay model.

### 2.7. Study Outcomes

The principal epidemiological outcomes were advanced-stage disease, stage IV disease, any recorded organ-specific metastasis, pulmonary resection among patients with stage I–II disease, sublobar resection rather than lobectomy among surgically treated patients with stage I–II disease, and treatment delay exceeding 90 days. The survival outcomes were all-cause mortality and lung cancer-specific mortality within 12 months of diagnosis. Overall survival time was calculated using the SEER survival-days variable. Death from any cause was considered an event, whereas patients alive at the last recorded follow-up were censored. For cancer-specific survival, death attributable to the index lung cancer diagnosis was considered the event. Patients who remained alive or died from another cause were censored, while those with a missing or unknown cause of death were excluded. In the competing-risk analyses, death from a cause other than lung cancer was treated as the competing event.

### 2.8. Statistical Analysis

Continuous variables were summarised using means and standard deviations (SDs), whereas the categorical variables were presented as frequencies and percentages. Baseline, tumour, and treatment characteristics were compared across the pre-COVID-19, COVID-19, and post-COVID-19 periods. Pearson’s chi-square test was used to compare categorical variables. Given the large sample size, statistical significance was interpreted together with the magnitude and clinical relevance of the observed differences.

Two interpretive conventions apply throughout. Estimates associated with the diagnostic period are adjusted associations between calendar period and outcome; because period is a proxy for exposure to healthcare disruption and the design is observational, they are not estimates of an effect of the pandemic. Shapley values reported for the prediction models describe the contribution of a variable to the model’s predictions and are not estimates of a causal effect on the outcome.

Separate multivariable logistic regression models were constructed for advanced-stage disease, stage IV disease, any organ-specific metastasis, pulmonary resection among patients with stage I–II disease, sublobar resection versus lobectomy among patients with stage I–II disease who underwent either procedure, and treatment delay exceeding 90 days. The pre-COVID-19 period served as the reference category. All models were adjusted for age at diagnosis, sex, marital status, median household income, and rurality. The sublobar-versus-lobectomy model was additionally adjusted for tumour-size category, whereas the treatment-delay model was additionally adjusted for the stage. Results were reported as adjusted odds ratios (OR) with 95% confidence intervals (CI). Missing outcome and covariate data were handled using complete-case analysis within each model.

Twelve-month survival analyses were restricted to patients diagnosed between 2018 and 2022, with follow-up administratively censored at 365 days. Cox proportional-hazards regression was initially used to evaluate overall and cancer-specific mortality. The models included diagnostic period, age, sex, marital status, median household income, rurality, and stage, and robust variance estimators were applied. The proportional-hazards assumption was evaluated using Schoenfeld residuals and global proportional-hazards tests. Because the assumption was violated for the COVID-period coefficient and selected stage categories, the final models were stratified by stage and marital status and included a time-varying COVID-period coefficient through an interaction with the natural logarithm of analysis time. COVID-versus-pre-COVID hazard ratios (HR) were therefore estimated at 30, 90, 180, and 365 days. The early post-COVID coefficient was modelled as time-invariant.

Fine-Gray subdistribution hazard regression was additionally used to evaluate 12-month lung cancer-specific mortality while treating death from other causes as a competing event [13]. The adjusted competing-risk model included age, sex, marital status, median household income, rurality, and stage. Results were reported as subdistribution HRs with 95% CIs. All statistical tests were two-sided, and *p* < 0.05 was considered statistically significant. Analyses were conducted using StataIC version 16, 64-bit (StataCorp LLC, College Station, TX, USA). Cause-specific Cox models treat death from other causes as censoring and therefore assume that such censoring is independent of the risk of cancer death. This assumption is questionable during a period in which an excess competing cause of death was present, which is why Fine-Gray subdistribution models were additionally fitted rather than reported as an alternative to the cause-specific analyses. Patients with a missing or unknown underlying cause of death were excluded from the cancer-specific analyses, which accounts for the difference in analysed sample size between the all-cause and cancer-specific models reported. Because the cohort size confers power to detect differences of negligible magnitude, every adjusted relative estimate is reported together with the observed proportion in each period and the absolute difference in percentage points, and statistical significance is not interpreted as evidence of clinical relevance in the absence of a material absolute difference.

### 2.9. Machine Learning Cohort, Outcome, and Predictor Eligibility

The epidemiological analyses described above characterise how presentation and treatment delivery changed across calendar periods at the population level. They do not address the question a clinician faces when meeting an individual patient at the point of diagnosis: how likely is this particular patient to die within the next year, and should they be prioritised for earlier supportive-care involvement, more intensive follow-up, or expedited multidisciplinary review? This question differs in kind rather than in scale. Period-level odds ratios describe average differences between groups and are not designed to generate an individual probability, whereas a prediction model estimates absolute risk for one patient from their own characteristics. Because our findings indicate that treatment delay persisted beyond the acute pandemic period, prioritisation under continuing capacity constraints is a practical problem rather than a hypothetical one. We therefore developed a prediction model with a deliberately narrow and clinically defined intended use: estimation of the probability of death within 12 months, computed at initial presentation, from information already documented in the diagnostic record at that moment. Flexible machine learning algorithms were included alongside penalised logistic regression, not on the assumption that they would outperform it but to establish whether any non-additive structure in these variables is left unexploited by conventional regression; the comparison is itself an informative result.

#### 2.9.1. Cohort and Outcome

The prediction analysis was designed to test whether 12-month mortality can be estimated at the moment of initial presentation using information routinely documented at diagnosis. Patients diagnosed with lung adenocarcinoma between 2018 and 2021 formed the model-development cohort (*n* = 87,281), and patients diagnosed during 2022 were held out as a temporal validation cohort within SEER (*n* = 22,012). This cohort took no part in model fitting, hyperparameter selection, model selection, or threshold derivation, and no recalibration was applied to it; it therefore provides an unbiased assessment of stability over calendar time within the same registry system, but not of transportability to other populations or health systems. Cases diagnosed in 2023 were excluded because 12-month follow-up was incomplete. The outcome was all-cause death within 12 months (365 days) of diagnosis. Patients with follow-up shorter than 12 months and no observed death were excluded to avoid outcome misclassification. Within the development cohort, 30,822 patients (35.3%) died within 12 months, compared with 7526 patients (34.2%) in the 2022 cohort. This exclusion reconciles the prediction cohorts with the epidemiological cohort as follows. Of the 87,879 patients diagnosed during 2018–2021, 598 were excluded for insufficient follow-up without an observed death, giving a development cohort of 87,281. Of the 45,427 patients diagnosed during 2022–2023, those diagnosed in 2023 were excluded in full because 12-month follow-up was unavailable, and a further small number of 2022 diagnoses were excluded under the same rule, giving a temporal-validation cohort of 22,012. No patient appeared in both cohorts, and the two cohorts shared no diagnosis years, so the temporal separation is complete. Analysis-specific populations and exclusions are summarised in Supplementary Table S2.

#### 2.9.2. Predictor Eligibility and the Time-of-Use Restriction

Because the intended use of the model is at initial presentation, every candidate variable was classified a priori according to whether its value is knowable at that time point. Variables recorded during the diagnostic work-up, including demographic and area-level socioeconomic characteristics, primary site, laterality, histology, diagnostic confirmation, grade, anatomical extent of disease, tumour size, organ-specific metastatic involvement, pleural invasion, and ipsilateral nodule status, were classified as available at diagnosis.

Five variables were classified as post-diagnosis and were excluded from the primary model: chemotherapy, radiotherapy, surgery type, reason for no surgery, and time from diagnosis to first treatment. These describe events that occur after the diagnostic encounter and after treatment decisions have been made. Time from diagnosis to first treatment additionally requires knowledge of a future event, so it cannot be observed at presentation by construction. Two further properties of these variables were established empirically in the development cohort and are reported here because they bear directly on the validity of any model that contains them.

1. One level of the reason-for-no-surgery variable records that the patient died before the recommended operation could be performed. All 116 development-cohort patients in this category died within 12 months, so this category encodes the outcome itself rather than predicting it;
2. Missingness of time from diagnosis to first treatment is strongly outcome-related: 12-month mortality was 73.0% when the value was missing and 26.8% when it was recorded. A missing value therefore functions as a marker of no treatment having been delivered, which is itself a consequence of early clinical deterioration.

Identifiers, the cohort label, diagnosis year, survival time, vital status, and cause-of-death fields were excluded from all models. Race and ethnicity were absent for the entire extract and could not be used as a predictor or examined as a fairness axis.

#### 2.9.3. Analysis Specifications

Three predictor specifications were pre-specified. Specification A is the primary model and contains only the variables available at diagnosis, using stage group as the measure of anatomical extent. Specification B is a pre-specified sensitivity analysis that is identical to A except that stage group is replaced by its T, N and M components. It was included because stage group and M category convey overlapping information, and entering both would divide variable attribution between collinear terms without adding independent signal. Specification C reproduces the previously reported predictor set, including the five post-diagnosis variables, and is reported solely to quantify how much apparent performance derives from information unavailable at presentation. Specification C is not a model that can be used at initial presentation and is labelled accordingly throughout. For the same reason, categorical duplicates of continuous measurements (tumour size category, treatment delay category) were not entered alongside their continuous counterparts. Throughout the manuscript, the results obtained under Specification C are identified as such in every table and figure in which they appear and are described as a reference analysis rather than as model performance.

#### 2.9.4. Data Preparation and Missing Data

Continuous predictors were median-imputed and standardised; categorical predictors were imputed with the modal category and one-hot encoded. All imputation and scaling parameters were estimated inside each training fold only, so that no validation or temporal-test information entered preprocessing. ICD-O-3 histology codes were handled as nominal categories rather than as a numeric quantity. Missingness was low for most predictors but reached 15.4% for tumour size, 11.1% for ipsilateral nodule status and 67.1% for pleural invasion. For several of these variables, missingness was itself outcome-related: 12-month mortality was 62.0% when the tumour size was unrecorded compared with 30.5% when it was recorded, and 47.0% versus 11.5% for pleural invasion, consistent with incomplete staging in patients too unwell for a full work-up. Single imputation cannot represent this mechanism, and no missingness indicators were entered in the reported analyses; the implications are considered in Section 4.

Because missingness in several staging variables was outcome-related, a prespecified sensitivity analysis repeated the primary specification with explicit missingness indicators retained. Continuous predictors received a binary indicator of missingness alongside the median-imputed value, and categorical predictors received an explicit “missing” level rather than being assigned the modal category, expanding the design matrix from 91 to 104 columns. Hyperparameters, cross-validation partitions, the locked threshold rule, and the random seed were unchanged, so the two analyses differ only in whether missingness was modelled or imputed away. Results are reported in Section 3.5 and Supplementary Table S11.

#### 2.9.5. Algorithms, Hyperparameter Tuning, and Internal Validation

Four algorithms representing distinct model families were fitted within each specification: L2-penalised logistic regression, random forest, gradient boosting, and a linear support vector machine (SVM) whose decision scores were converted to probabilities by Platt scaling inside a cross-validated wrapper. Hyperparameters were selected by exhaustive grid search over a pre-specified search space, using three-fold stratified cross-validation on a stratified random subsample of 30,000 development-cohort patients with the area under the receiver operating characteristic curve as the objective. The complete search space and the values selected for every algorithm and specification are given in Supplementary Table S7. Under the primary specification, the selected values were an inverse regularisation strength of 0.1 for logistic regression; 300 trees of unlimited depth with a minimum of 5 observations per leaf for the random forest; 400 boosting stages with a learning rate of 0.05, a maximum depth of 3 and a subsampling fraction of 0.8 for gradient boosting; and an inverse regularisation strength of 0.01 for the SVM. The random seed was fixed at 42 for all fitting and resampling; no other settings were tuned.

Reported development-cohort performance is not apparent performance. It was obtained by five-fold stratified cross-validation of the whole development cohort, with the out-of-fold predicted probabilities pooled across folds, so every development estimate is internally validated. The final model for each algorithm was then refitted once on the entire development cohort using the selected hyperparameters and applied to the 2022 cohort without any refitting, recalibration or threshold re-estimation. This design provides temporal validation within a single registry system and is therefore a test of stability over calendar time rather than of transportability to other populations.

The outcome prevalence of approximately 35% does not constitute severe class imbalance, and no over-sampling, under-sampling or synthetic minority oversampling was applied. This was deliberate: imbalance corrections distort predicted probabilities and would have invalidated the calibration analysis described below.

#### 2.9.6. Model Output and Decision Threshold

The output of each model is a predicted probability of death within 12 months. For the binary summaries, a single operating point was required. The selection rule was fixed in advance as the point maximising the Youden index, and it was applied to the pooled development out-of-fold predictions only. The resulting numeric threshold was then locked and applied unchanged to the 2022 cohort, so that the reported temporal-validation classification metrics contain no threshold optimisation on the validation data. Five alternative rules (closest point to the top-left corner of the receiver operating characteristic curve, the point achieving at least 85% sensitivity, the prevalence-matched threshold, the F1-maximising threshold, and a fixed 0.50) were evaluated for transparency and are tabulated in Supplementary Data S1. Throughout, a patient is described as flagged when the predicted probability equals or exceeds the locked threshold. Flagging is a classification of a patient, not a detection of an event; statements that the model identified a given number of deaths mean that the corresponding number of patients who subsequently died within 12 months were flagged at diagnosis. The term clinical utility is used in its decision-analytic sense, denoting net benefit relative to the strategies of acting on all patients or on none, and not as a general claim of usefulness in practice.

#### 2.9.7. Performance Measures

Discrimination was summarised by the area under the receiver operating characteristic curve (ROC AUC) and the area under the precision–recall curve (PR AUC) [14]. Calibration was assessed by the calibration intercept (calibration in the large, estimated with the linear predictor as an offset), the calibration slope, the Brier score, the scaled Brier score relative to a non-informative model with the observed prevalence, and flexible loess-smoothed calibration curves with decile groups and 95% Wilson intervals [15]. Clinical utility was assessed by decision curve analysis, in which net benefit is compared with the strategies of acting on every patient and on no patient across a range of threshold probabilities [16]. All continuous estimates carry percentile bootstrap 95% CIs based on 500 resamples. Correlated areas under the curve, which arise when specifications or algorithms are compared in the same 2022 participants, were compared with DeLong’s test for paired receiver operating characteristic curves [17], and the paired difference between the two best-performing algorithms was additionally estimated by bootstrap resampling.

#### 2.9.8. Interpretability

Variable attribution was computed with Shapley additive explanations (SHAP) [18]. Exact TreeSHAP was used for the tree ensembles and LinearSHAP for logistic regression. Attributions were computed from a single model fitted on the development cohort and applied unchanged to both cohorts; the model was never refitted on the 2022 data for this purpose. One-hot columns belonging to the same source variable were summed per patient before importance was computed, which is the correct aggregation for additive attributions and prevents a variable’s importance from being divided among its own indicator columns.

#### 2.9.9. Software

Analyses were performed in Python 3.13.15 in the Google Colaboratory environment (Google LLC, Mountain View, CA, USA), with the reported results generated in a single execution on 30 August 2026. Model fitting, cross-validation, hyperparameter search, and the computation of discrimination and Brier statistics used scikit-learn 1.6.1; Shapley additive explanations used shap 0.52.0; and data handling used NumPy 2.1.3 and pandas 2.2.3.

The calibration intercept and slope, the expected calibration error, the decision-curve net benefit, and DeLong’s test for correlated areas under the curve were implemented directly in the analysis script rather than obtained from a third-party package, and these implementations are contained in the deposited code; results depending on them are therefore reproducible independently of library version.

The random seed was fixed at 42 for model fitting, cross-validation partitioning, bootstrap resampling, and the sampling used for Shapley additive explanations. The script records the software versions, every configuration parameter, the random seed, and the execution timestamp at run time and writes them to a machine-readable configuration record included with the results (Supplementary Data S1), so that the environment described here can be verified against the environment that produced the results. The complete analysis script is available as Supplementary Software S1.

#### 2.9.10. Comparison with Stage-Based Risk Classification

To express model performance in terms comparable to current practice, predictions in the temporal-validation cohort were compared with two rules based on stage alone: classification of all patients with stage IV disease as high risk and classification of all patients with stage III or IV disease as high risk. Patients whose stage group was not recorded (*n* = 743) were treated as not meeting either rule, reflecting how such a patient would be handled if stage were the only available criterion. Sensitivity, specificity, predictive values, and the absolute numbers of patients flagged and deaths identified were computed for each rule and for the model at its locked threshold in the same 22,012 participants. This comparison was not pre-specified; it was added during revision in response to peer review and is reported as a post hoc analysis.

### 2.10. Ethical Considerations

SEER provides deidentified, publicly available cancer-registry data and contains no directly identifiable patient information. The present study involved secondary analysis of deidentified data and no direct patient contact. Therefore, individual informed consent was not required. Institutional review board approval was not required for this analysis, subject to applicable institutional policies and compliance with the SEER Data Use Agreement.

## 3. Results

### 3.1. Baseline Characteristics

A total of 133,306 patients with lung adenocarcinoma were included, comprising 45,873 patients diagnosed during the pre-COVID period (2018–2019), 42,006 during the COVID-19 period (2020–2021), and 45,427 during the post-COVID period (2022–2023). The mean age at diagnosis was 70.0 years (SD, 10.3), with a modest increase from 69.8 years before COVID-19 to 70.4 years after COVID-19 (*p* < 0.001). Overall, 54.3% of patients were female, 51.9% were married or partnered, and 60.3% resided in large metropolitan areas. Although distributions of age, sex, marital status, household income, primary tumour site, laterality, tumour grade, and diagnostic confirmation differed significantly across the three periods, rurality did not differ significantly (*p* = 0.074). The proportion of diagnoses confirmed by positive histology increased from 89.3% before COVID-19 to 90.2% during COVID-19 and 91.2% after COVID-19 (*p* < 0.001) (Table 2).

### 3.2. Tumour Characteristics and Treatment Patterns

The distribution of stage at diagnosis differed significantly across the diagnostic periods (*p* < 0.001). Stage IV disease was identified in 45.7% of patients before COVID-19, 46.3% during COVID-19, and 42.8% during post-COVID-19 (2022–2023). Correspondingly, advanced-stage disease (stage III–IV) accounted for 60.0%, 59.9%, and 56.5% of diagnoses, respectively. The proportion of patients with an unknown stage remained stable across the three periods (3.5%, 3.3%, and 3.5%; *p* = 0.340).

Any recorded organ-specific metastasis was present in 43.8% of patients before COVID-19, increased to 45.4% during COVID-19, and decreased to 42.6% after COVID-19 (*p* < 0.001). Bone and brain metastases were slightly more frequent during the COVID-19 period, whereas lung metastases were less frequent in the post-COVID period. Tumour-size distribution and the presence of separate ipsilateral tumour nodules also differed significantly across periods (both *p* < 0.001).

Primary-site surgery was performed in 29.4% of patients before COVID-19, 29.2% during COVID-19, and 30.4% after COVID-19 (*p* < 0.001). Pulmonary resection rates were 29.2%, 28.9%, and 30.1%, respectively. The proportion undergoing sublobar resection increased from 7.2% in both the pre-COVID and COVID-19 periods to 8.4% after COVID-19. In contrast, radiotherapy use declined progressively from 37.4% before COVID-19 to 36.2% during COVID-19 and 34.7% after COVID-19 (*p* < 0.001), whereas chemotherapy use remained relatively stable (39.8%, 39.9%, and 39.3%; *p* = 0.160).

Treatment intervals also differed significantly across periods (*p* < 0.001). The proportion treated on the day of diagnosis decreased from 11.9% before COVID-19 to 10.3% during COVID-19 and 7.5% after COVID-19. Conversely, treatment delays exceeding 90 days increased from 8.3% to 9.0% and 11.7%, respectively (Supplementary Table S4). The complete tumour and treatment distributions are provided in Supplementary Table S4.

### 3.3. Adjusted Associations with Stage, Metastasis, and Treatment

After adjustment for demographic and socioeconomic characteristics, the COVID-19 period was not associated with the overall likelihood of advanced-stage disease compared with the pre-COVID-19 period (61.9% versus 62.1%; absolute difference, −0.2 percentage points; OR, 1.00; 95% CI, 0.98–1.03; *p* = 0.838). COVID-era diagnoses had slightly higher odds of stage IV disease (47.9% versus 47.4%; +0.5 percentage points, equivalent to 5 additional patients per 1000; OR, 1.03; 95% CI, 1.00–1.06; *p* = 0.030) and of any organ-specific metastasis (45.8% versus 44.4%; +1.4 percentage points, or 14 per 1000; OR, 1.06; 95% CI, 1.04–1.09; *p* < 0.001). Although both estimates reached statistical significance, the absolute differences are of a magnitude unlikely to be perceptible in clinical practice.

Among patients with stage I–II disease, the COVID-19 period was not associated with pulmonary resection (64.0% versus 64.1%; −0.1 percentage points; OR, 0.99; 95% CI, 0.94–1.04; *p* = 0.660), and no significant difference was found in the odds of sublobar resection rather than lobectomy (OR, 1.02; 95% CI, 0.95–1.09; *p* = 0.647). The odds of treatment delay exceeding 90 days were higher during COVID-19 than before the pandemic (11.1% versus 10.2%; +0.9 percentage points, or 9 per 1000; OR, 1.11; 95% CI, 1.06–1.17; *p* < 0.001).

**Figure 2:**
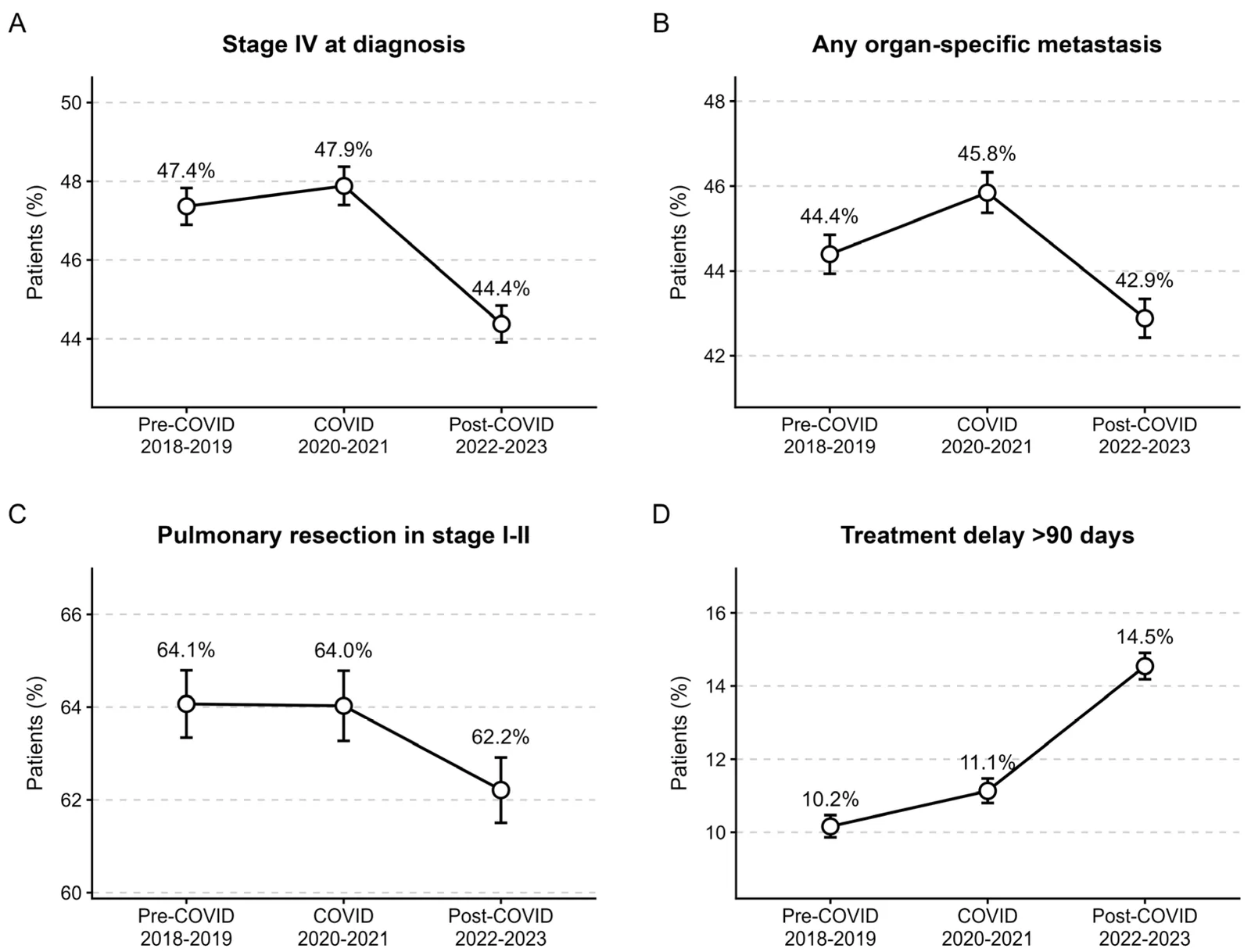
Clinical outcomes across the COVID-19 diagnostic periods: pre-COVID-19 (2018–2019), COVID-19 (2020–2021), and post-COVID-19 (2022–2023). (**A**) Stage IV disease at diagnosis; (**B**) any recorded organ-specific metastasis; (**C**) pulmonary resection among patients with stage I–II disease; and (**D**) treatment delay exceeding 90 days. Circles represent observed proportions, and vertical range-lines indicate 95% Wilson confidence intervals. Patients with missing outcome-specific data were excluded.

Compared with the pre-COVID-19 period, post-COVID-19 (2022–2023) diagnoses were associated with lower odds of advanced-stage disease (58.5% versus 62.1%; −3.6 percentage points; OR, 0.88; 95% CI, 0.85–0.90), stage IV disease (44.4% versus 47.4%; −3.0 percentage points; OR, 0.90; 95% CI, 0.88–0.93), and any organ-specific metastasis (42.9% versus 44.4%; −1.5 percentage points; OR, 0.95; 95% CI, 0.92–0.98; all *p* < 0.001). Patients diagnosed after COVID-19 had lower odds of pulmonary resection for stage I–II disease (62.2% versus 64.1%; −1.9 percentage points; OR, 0.92; 95% CI, 0.88–0.96; *p* = 0.001), higher odds of sublobar resection rather than lobectomy (OR, 1.20; 95% CI, 1.13–1.28; *p* < 0.001), and substantially higher odds of treatment delay exceeding 90 days (14.5% versus 10.2%; +4.3 percentage points, equivalent to 43 additional patients per 1000; OR, 1.45; 95% CI, 1.39–1.52; *p* < 0.001) (Table 3 and Figure 2). The treatment-delay finding is the only estimate in this analysis whose absolute magnitude is of a scale likely to be apparent at the service level.

All models were adjusted for age, sex, marital status, median household income, and rurality, and used complete-case analysis. The analysed N for each model is shown in the final column and reconciles with the full cohort of 133,306 patients as follows.

Advanced stage (III–IV) and stage IV. Because stage was the dependent variable, the 4551 patients with unknown stage were excluded, leaving 128,755 with known stage; a further 100 were excluded for missing model covariates, giving 128,655.

Any organ-specific metastasis. A total of 1349 patients were excluded because metastatic status could not be determined from the component site variables or because model covariates were missing, giving 131,957.

Pulmonary resection in stage I–II. The model was restricted to the 50,457 patients with stage I–II disease, of whom 81 were excluded for missing the surgical outcome or covariates, giving 50,376.

Sublobar resection versus lobectomy. The model was further restricted to patients with stage I–II disease who underwent either sublobar resection or lobectomy and had complete covariate and tumour-size data, giving 31,299. Local tumour destruction, pneumonectomy, extended procedures, and resection not otherwise specified are not part of this comparison.

Treatment delay exceeding 90 days. A total of 26,743 patients were excluded. This comprises 25,129 with unknown treatment timing and 4551 with unknown stage, since the model was adjusted for stage; 2937 patients met both criteria and are counted once, which accounts for the difference between the sum of the two groups and the total excluded.

The sublobar-versus-lobectomy model was additionally adjusted for tumour size and the treatment-delay model for stage. Percentages reported in the text are calculated on the corresponding analysed population rather than on the full cohort.

Observed proportions are the unadjusted values within the population analysed by the corresponding model and are plotted in Figure 2. Absolute differences are expressed in percentage points (pp) and are unadjusted; because the adjustment covariates were similarly distributed across periods, adjusted marginal differences would be expected to differ only slightly. Observed proportions are not given for the sublobar-versus-lobectomy comparison because its model population, patients with stage I–II disease undergoing either procedure, is not a reporting stratum in the descriptive tables; among all patients undergoing either procedure irrespective of stage, sublobar resection accounted for 25.6%, 25.8%, and 28.4% of the three periods, respectively. Analysis-specific populations are also summarised in Supplementary Table S2. OR is odds ratio, and CI, confidence interval.

### 3.4. Twelve-Month Mortality

Because the proportional-hazards assumption was violated for the COVID-period coefficient, the association between the diagnostic period and mortality was estimated at prespecified follow-up times. In the adjusted time-varying Cox model for overall mortality, patients diagnosed during COVID-19 had a higher mortality hazard at 30 days than those diagnosed before COVID-19 (HR, 1.05; 95% CI, 1.02–1.08; *p* = 0.002). The association was no longer significant at 90 days (HR, 1.01; 95% CI, 0.98–1.03; *p* = 0.612) or 180 days (HR, 0.98; 95% CI, 0.95–1.01; *p* = 0.144). At 365 days, the estimated hazard was lower during COVID-19 (HR, 0.95; 95% CI, 0.92–0.99; *p* = 0.012). Early post-COVID diagnosis in 2022 was not significantly associated with overall mortality (HR, 0.99; 95% CI, 0.96–1.02; *p* = 0.498). For cancer-specific mortality, no significant COVID-era difference was found at 30 days (HR, 1.02; 95% CI, 0.99–1.06; *p* = 0.202). Lower hazards were observed at 90 days (HR, 0.97; 95% CI, 0.95–1.00; *p* = 0.032), 180 days (HR, 0.94; 95% CI, 0.91–0.97; *p* < 0.001), and 365 days (HR, 0.91; 95% CI, 0.87–0.95; *p* < 0.001). Early post-COVID diagnosis was also associated with lower cancer-specific mortality (HR, 0.96; 95% CI, 0.93–0.99; *p* = 0.011).

In the adjusted Fine-Gray competing-risk analysis, with other-cause death treated as the competing event, the cumulative incidence of lung cancer death was lower among patients diagnosed during COVID-19 (sHR, 0.96; 95% CI, 0.94–0.99; *p* = 0.003) and during early post-COVID 2022 (sHR, 0.95; 95% CI, 0.92–0.98; *p* = 0.001) than among those diagnosed before COVID-19 (Table 4). The subdistribution estimate at 365 days was closer to the null than the corresponding cause-specific estimate (sHR, 0.96 versus HR, 0.91), and the cancer-specific analyses included 787 fewer patients than the all-cause analyses because of missing or unknown cause of death.

### 3.5. Model Discrimination and Calibration

**Figure 3:**
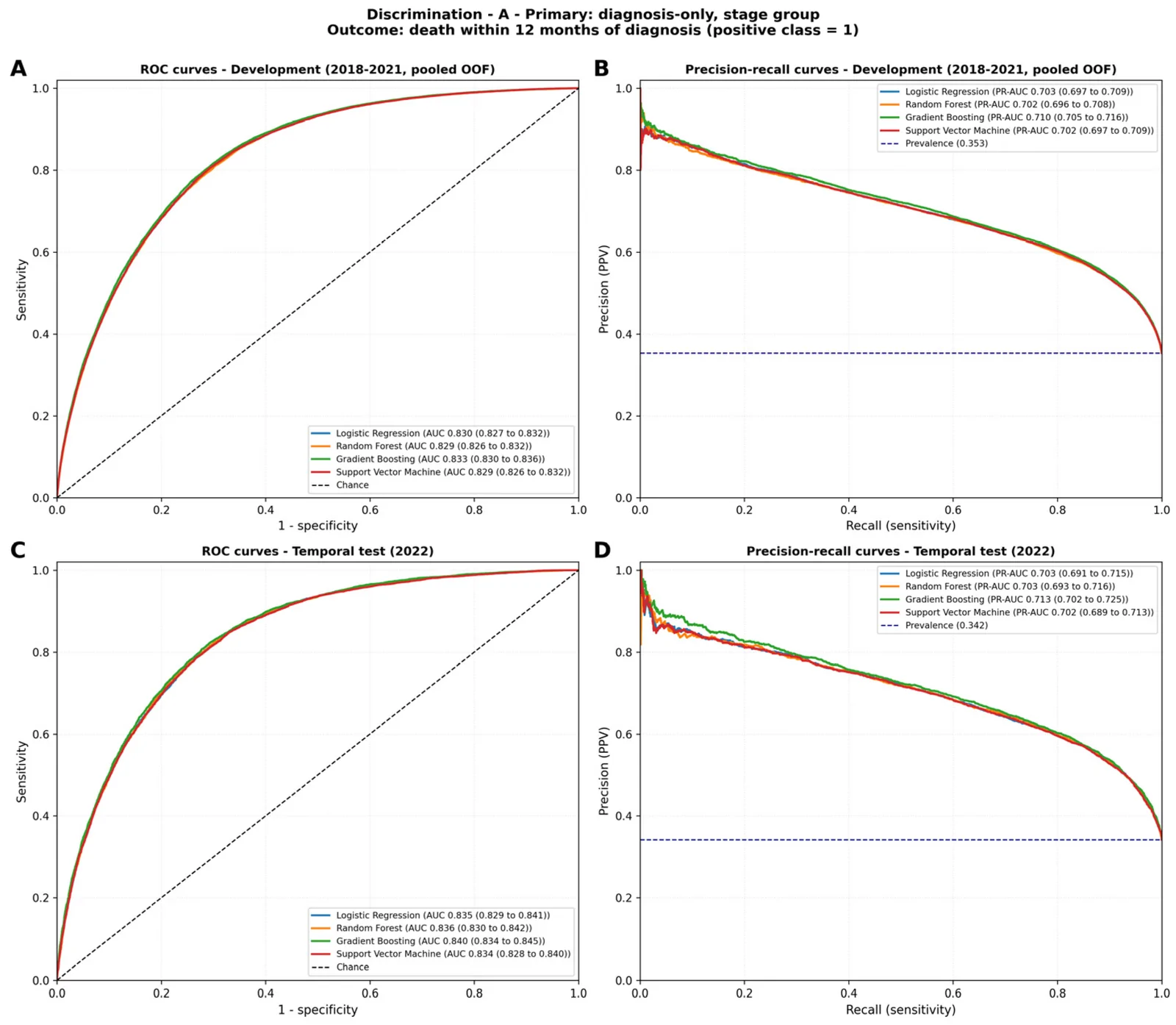
Discrimination of the diagnosis-only models (Specification A). (**A**) Receiver operating characteristic curves, development cohort (pooled out-of-fold predictions); (**B**) precision–recall curves, development cohort; (**C**) receiver operating characteristic curves, 2022 temporal validation cohort; (**D**) precision–recall curves, 2022 temporal validation cohort. Dashed lines denote chance performance in the receiver operating characteristic panels and the outcome prevalence in the precision–recall panels.

The model-development cohort comprised 87,281 patients diagnosed during 2018–2021, of whom 30,822 (35.3%) died within 12 months. The held-out temporal-validation cohort within SEER comprised 22,012 patients diagnosed during 2022, including 7526 deaths (34.2%). Under the primary diagnosis-only specification, pooled out-of-fold ROC AUCs in the development cohort ranged from 0.829 to 0.833, whereas ROC AUCs in the 2022 temporal-validation cohort ranged from 0.834 to 0.840. Gradient boosting achieved the highest temporal-validation ROC AUC (0.840; 95% CI, 0.834–0.845) and PR AUC (0.713; 95% CI, 0.702–0.725), although the performance differences between algorithms were small (Table 5 and Figure 3). Because PR AUC is prevalence-dependent, with a non-informative value equal to the outcome prevalence, PR AUC values are reported alongside the prevalence of the corresponding cohort in Table 5 and are not directly comparable between cohorts. For gradient boosting, the PR AUC was 0.710 against a prevalence of 0.353 in development and 0.713 against 0.342 in temporal validation, so the margin above the non-informative baseline was 0.357 and 0.371, respectively.

The difference between the best- and worst-performing algorithms in the 2022 cohort was 0.006 ROC AUC units. The paired difference between gradient boosting and penalised logistic regression, estimated in the same participants, was 0.0046 (95% CI, 0.0035 to 0.0058; DeLong *p* < 0.001), with an identical estimate and interval from 2000 bootstrap resamples. Although statistically significant in a validation cohort of 22,012 patients, the upper confidence limit excludes any difference exceeding 0.006 ROC AUC units, and the predicted risks from the two models correlated at a Spearman coefficient of 0.988. The corresponding differences were 0.010 for PR AUC (0.713 versus 0.703) and 0.0021 for the Brier score (0.1527 versus 0.1548). Inner cross-validated ROC AUC at the selected hyperparameters spanned only 0.004 across the four algorithm families (0.8263 to 0.8301; Supplementary Table S7), indicating that the ranking was not an artefact of tuning.

**Figure 4:**
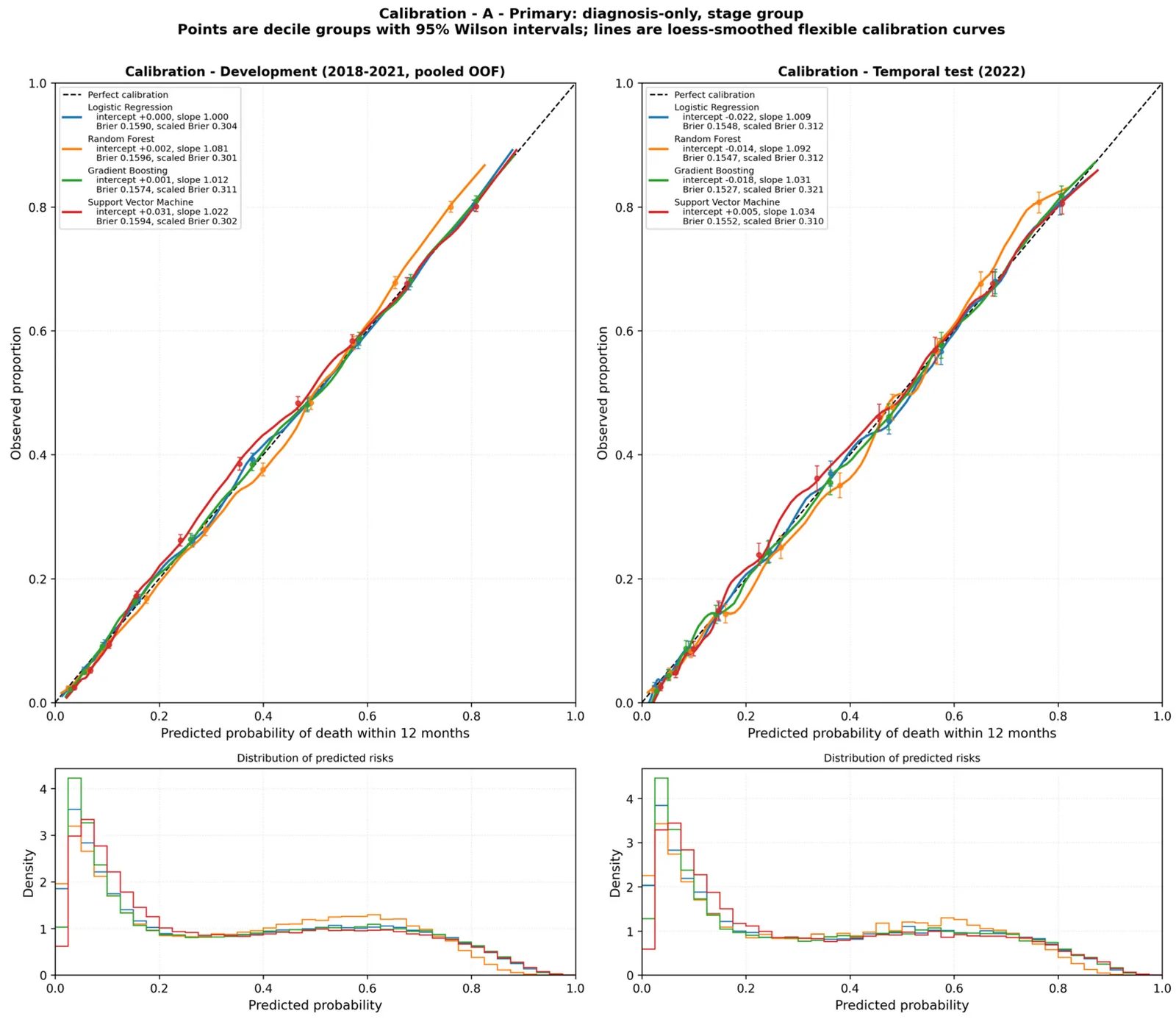
Calibration of the diagnosis-only models (Specification A) in the development cohort (**left**) and the 2022 temporal validation cohort (**right**). Colours distinguish the four machine learning algorithms as indicated in the legend (blue: Logistic Regression, orange: Random Forest, green: Gradient Boosting, red: Support Vector Machine). Dashed lines indicate reference baselines (black: chance/perfect calibration; blue: prevalence). Points represent decile groups of predicted risk with vertical range-lines indicating 95% Wilson confidence intervals; lines are loess-smoothed flexible calibration curves; the dashed diagonal denotes perfect calibration. Calibration intercept, calibration slope, Brier score, and scaled Brier score are given in the legend of each panel. The lower panels show the distribution of predicted probabilities by model algorithm. No recalibration was applied to the 2022 cohort.

Calibration was maintained in the temporal-validation cohort without recalibration. For gradient boosting, the calibration intercept was −0.018 (95% CI, −0.053 to 0.015), indicating no systematic over- or under-estimation of overall risk, and the mean predicted risk closely matched observed mortality (0.345 versus 0.342; observed-to-expected ratio, 0.992). The calibration slope was 1.031 (95% CI, 1.001 to 1.059); the interval marginally excluded unity, indicating slight compression of predicted risk toward the centre of the distribution, although the magnitude of this departure was small. The Brier score was 0.1527 (95% CI, 0.1500 to 0.1553) and the expected calibration error across ten equal-sized groups was 0.0058. Penalised logistic regression showed a calibration intercept of −0.022 (95% CI, −0.056 to 0.011) and a slope of 1.009 (95% CI, 0.980 to 1.038) and was the only model whose slope interval contained unity; the corresponding intervals were 1.031 (1.001 to 1.059) for gradient boosting, 1.034 (1.004 to 1.062) for the support vector machine, and 1.092 (1.059 to 1.125) for random forest. In the development cohort, the out-of-fold calibration was essentially perfect for gradient boosting (intercept, 0.001 [95% CI, −0.015 to 0.019]; slope, 1.012 [95% CI, 0.997 to 1.026]; expected calibration error, 0.0044). The flexible calibration curves remained close to the identity line across the predicted-risk range in both cohorts (Figure 4). The complete hyperparameter search space and selected configurations are reported in Supplementary Table S7.

In the prespecified sensitivity analysis retaining explicit missingness indicators, temporal-validation discrimination improved consistently but slightly across all four algorithms, by between 0.0036 and 0.0048 ROC AUC units (gradient boosting 0.843 versus 0.840; logistic regression 0.840 versus 0.835). Brier scores fell by 0.0015 to 0.0020, and calibration was essentially unchanged: for gradient boosting, the calibration intercept moved from −0.019 to −0.020 and the slope from 1.031 to 1.035. The direction and the magnitude of these differences are consistent with the interpretation that missingness in the staging variables carries prognostic information and that excluding it attenuates rather than inflates the reported performance (Supplementary Table S11).

Development estimates are pooled out-of-fold predictions from five-fold stratified cross-validation and are therefore internally validated; apparent performance is not reported. Temporal validation estimates were obtained by refitting each model once on the whole development cohort and applying it to the 2022 cohort without refitting, recalibration or threshold re-estimation. CIs are percentile bootstrap intervals from 500 resamples. For the development cohort, *n* = 87,281, 30,822 deaths within 12 months (35.3%). For the temporal validation cohort, *n* = 22,012, 7526 deaths (34.2%). A calibration intercept of zero and a calibration slope of one denote perfect calibration; a slope above one indicates predictions that are too conservative. The scaled Brier score expresses the reduction in squared prediction error relative to a non-informative model predicting the observed prevalence for every patient. ROC AUC, area under the receiver operating characteristic curve; PR AUC, area under the precision–recall curve.

### 3.6. Classification at the Locked Threshold and Net Benefit

**Figure 5:**
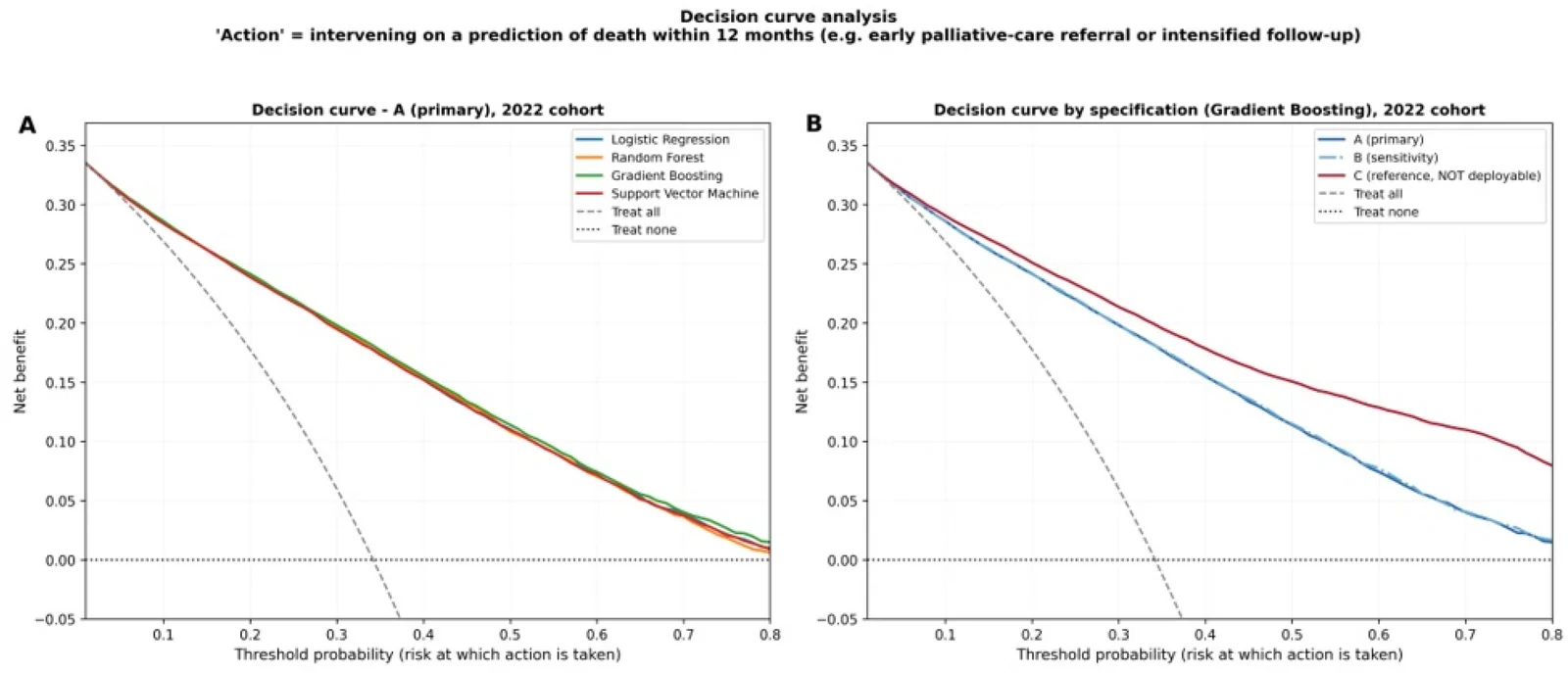
Decision curve analysis in the 2022 temporal validation cohort. The action modelled is intervening on a prediction of death within 12 months, such as earlier palliative-care referral or intensified follow-up. (**A**) All four algorithms under the primary diagnosis-only specification, compared with the treat-all and treat-none strategies. (**B**) Net benefit of the gradient boosting model under each of the three predictor specifications. The apparently greater net benefit of Specification C depends on treatment-course information that is unavailable when the decision would be made and does not represent attainable clinical utility.

At the locked threshold derived from the development out-of-fold predictions, gradient boosting achieved a sensitivity of 0.816, specificity of 0.712, positive predictive value of 0.596, negative predictive value of 0.882, F1 score of 0.689, and Matthews correlation coefficient of 0.503 in the 2022 cohort (Supplementary Table S5 and Supplementary Figure S1). The other algorithms produced similar classification performance. At their respective locked thresholds, gradient boosting classified 10,309 patients as high risk and correctly flagged 6143 patients who died, whereas penalised logistic regression classified 10,246 as high risk and correctly flagged 6069 patients who died. Gradient boosting therefore flagged 74 additional patients who died with 11 fewer false positives, equivalent to 3.4 additional deaths flagged and 0.5 fewer false alarms per 1000 patients. In the decision-curve analysis, all four diagnosis-only models showed positive net benefit across the evaluated threshold range and exceeded the treat-all strategy from a threshold probability of approximately 0.10 upward (Figure 5). The differences between algorithms were small at every threshold examined. The net-benefit advantage of gradient boosting over penalised logistic regression ranged from 0.0013 to 0.0036 across threshold probabilities from 0.10 to 0.50, equivalent to between 1.3 and 3.6 additional true positives per 1000 patients.

### 3.7. Incremental Value over Stage-Based Risk Classification

To express model performance in terms relevant to practice, predictions in the 2022 cohort were translated into absolute patient numbers and compared with classification by stage alone, which approximates how prognostic risk is currently stratified at presentation.

Predicted risk separated the cohort substantially and agreed closely with the observed outcomes across the whole range. Observed 12-month mortality rose from 1.9% (95% CI, 1.4–2.5) in the lowest decile of predicted risk to 81.8% (95% CI, 80.1–83.3) in the highest, a 43.9-fold gradient across deciles containing approximately 2200 patients each. Predicted and observed risk differed by no more than 1.3 percentage points in any decile, and the mean predicted value fell within the 95% CI of the observed proportion in nine of the ten deciles; the exception was the lowest decile, in which a predicted risk of 2.8% slightly exceeded the observed 1.9% (Supplementary Table S8).

Stage alone, entered as an ordinal variable, achieved a c-statistic of 0.779 in the 2022 cohort, against 0.840 for the model, an increment of 0.061. Classifying all 9692 patients with stage IV disease as high risk flagged 44.0% of the cohort and correctly flagged 5676 of the 7526 patients who died within 12 months (sensitivity, 0.754; specificity, 0.723; positive predictive value, 0.586; negative predictive value, 0.850), leaving 1850 who died unflagged. At its locked threshold, the gradient-boosting model flagged 10,309 patients (46.8%) and correctly flagged 6143 who died (sensitivity, 0.816; specificity, 0.712; positive predictive value, 0.596; negative predictive value, 0.882), leaving 1383 unflagged. The model therefore flagged 617 additional patients, of whom 467 died within 12 months and 150 did not, and reduced the number of deaths not flagged at diagnosis by 25.2%. Positive predictive value was marginally higher for the model (0.596 versus 0.586), so the additional detection was not obtained by accepting a less precise rule. Extending stage-based classification to include stage III disease raised sensitivity to 0.864 but flagged 58.1% of the cohort and reduced positive predictive value to 0.509.

In absolute terms, applying the model to 1000 consecutive patients at diagnosis would flag 468, of whom 279 of the 342 who die within 12 months would be correctly flagged, 189 who survive the year would be flagged unnecessarily, and 63 deaths would occur among patients not flagged. Stage-based classification applied to the same 1000 patients would flag 440, correctly flagging 258 deaths, flagging 182 survivors unnecessarily, and leaving 84 deaths unflagged. The model therefore flags 28 additional patients per 1000, of whom 21 die within the year and 7 do not.

### 3.8. Reference Specifications: Effect of Post-Diagnosis Variables and of T/N/M Representation

**Figure 6:**
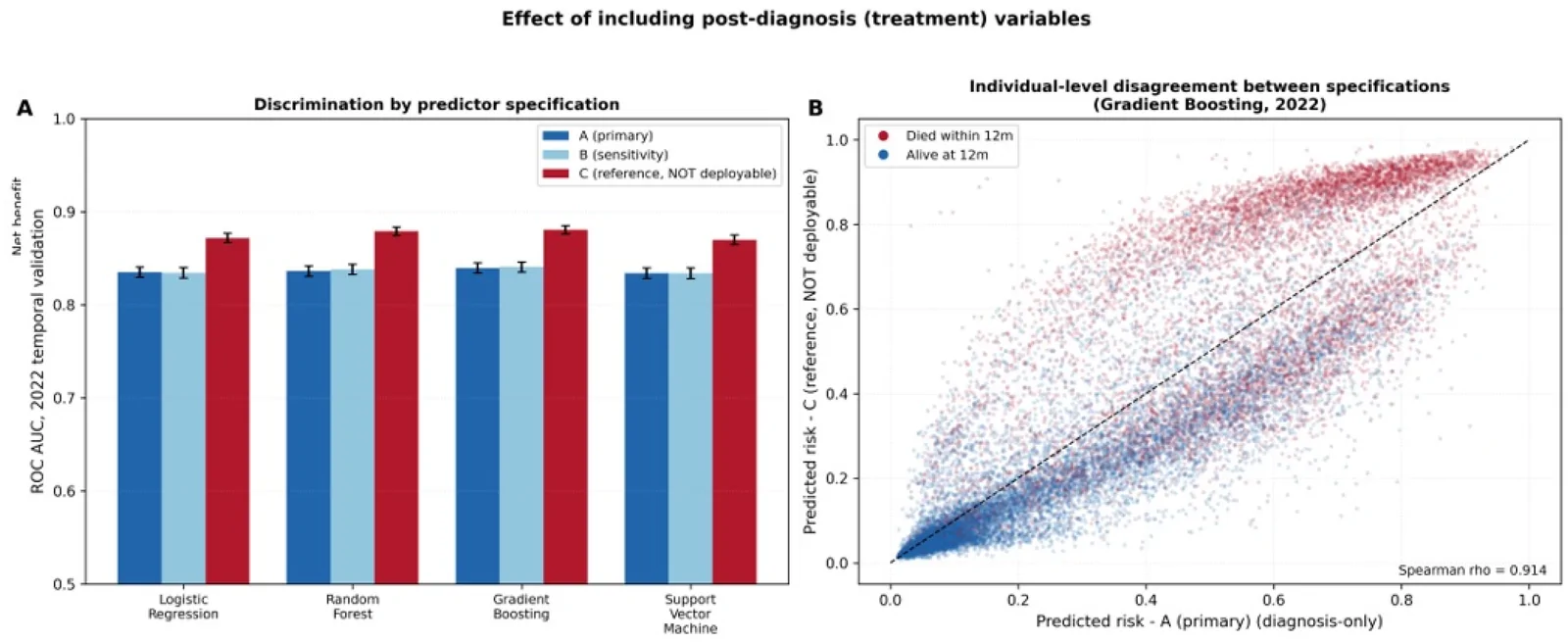
Effect of including post-diagnosis treatment variables. (**A**) Area under the receiver operating characteristic curve in the 2022 temporal validation cohort for each algorithm under the three predictor specifications; bars represent point estimates and vertical range-lines indicate percentile bootstrap 95% CIs. (**B**) Patient-level agreement between the predicted risks of the primary diagnosis-only specification and the reference specification containing post-diagnosis variables (gradient boosting, 2022 cohort); each point is one patient, coloured by observed 12-month outcome, and the dashed line is the line of identity. Divergence above the line indicates patients whose risk is raised by information the reference model has already observed and a clinician at diagnosis would not.

The analyses in this section are reported to quantify the consequences of two predictor choices and do not describe models usable at initial presentation. Specification C in particular contains information generated after the diagnostic encounter and, in one category, the outcome itself; the values below therefore measure retrospective outcome explanation rather than prediction at diagnosis. Including chemotherapy, radiotherapy, surgery type, reason for no surgery, and treatment delay increased temporal-validation ROC AUCs by 0.036–0.043 compared with the diagnosis-only specification (Table 6 and Table S6, and Figure 6). For gradient boosting, the ROC AUC increased from 0.840 to 0.880 and the PR AUC from 0.713 to 0.810. By contrast, replacing the overall stage group with separate T, N, and M components changed the ROC AUC by no more than 0.0021 across algorithms, indicating negligible differences in discrimination between the two diagnosis-only representations.

The receiver operating characteristic and precision–recall curves for Specification B were closely superimposed on those of Specification A in both the development and the 2022 cohorts, with all four algorithms falling within a narrow band (Supplementary Figure S3). At the threshold locked independently within each specification, the distribution of true and false classifications was similar in the development and 2022 cohorts for both Specification B and Specification C, and the higher specificity and positive predictive value seen under Specification C reflect the post-diagnosis information it contains rather than better prognostication at presentation (Supplementary Figure S8). Additional calibration and attribution analyses for Specifications B and C are presented in Supplementary Figures S4–S7.

### 3.9. Model Interpretation

**Figure 7:**
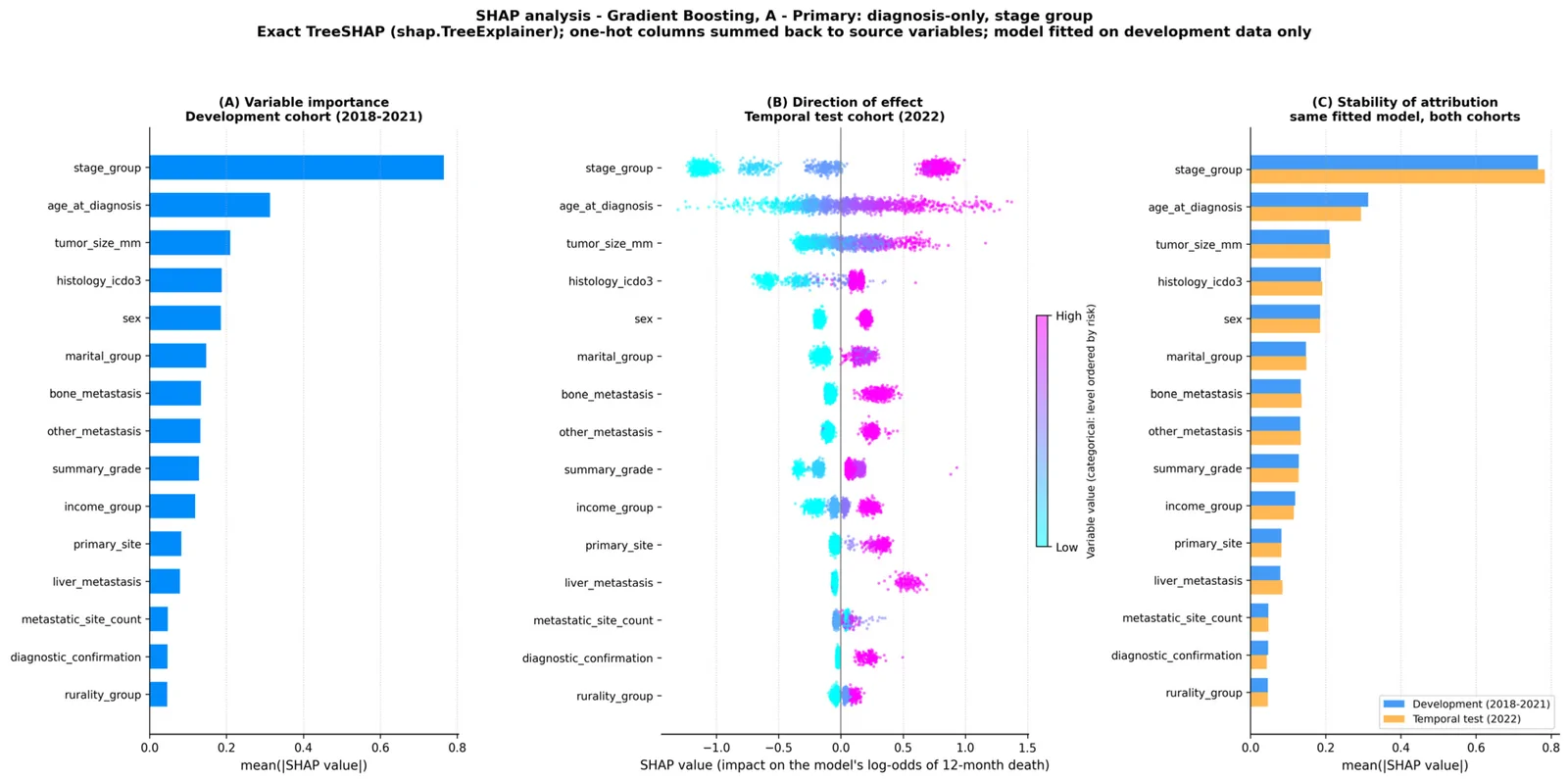
SHAP for the primary diagnosis-only gradient boosting model. (**A**) Mean absolute Shapley value per variable in the development cohort. (**B**) Direction and magnitude of individual attributions in the 2022 temporal validation cohort; each point is one patient, coloured by the value of the variable, with categorical levels ordered by observed risk. The solid vertical line indicates zero impact on the model’s log-odds, and light dashed lines across panels provide reference grid-lines. (**C**) Comparison of mean absolute Shapley values between the two cohorts using the same fitted model. Attributions were computed by exact TreeSHAP from a model fitted on development data only, and indicator columns were summed back to their source variable before importance was computed. Shapley values quantify each variable’s contribution to the model’s predicted probability and do not represent causal effects on mortality.

In the primary gradient-boosting model, stage group made the largest contribution to predicted 12-month mortality, followed by age at diagnosis, tumour size, histology, and sex. Bone, liver, and other-site metastases also shifted predictions toward higher mortality, and the ranking of contributions was stable between the development and temporal-validation cohorts (Figure 7). When post-diagnosis variables were included, chemotherapy, surgery type, treatment delay, reason for no surgery, and radiotherapy received a substantial share of the model’s attribution, and the baseline prognostic variables fell in the importance ranking (Supplementary Figure S2).

## 4. Discussion

In this population-based cohort of 133,306 adults with microscopically confirmed lung adenocarcinoma, the COVID-19 period was not associated with a uniform shift from stage I–II to stage III–IV disease after adjustment for demographic and area-level socioeconomic characteristics. Nevertheless, modest increases were observed in stage IV disease, organ-specific metastasis, and treatment initiation more than 90 days after diagnosis. During 2022–2023, stage distribution was more favourable, but treatment delays persisted and surgical management changed, with lower odds of pulmonary resection in stage I–II disease and a greater use of sublobar resection. These findings are consistent with diagnostic and treatment pathways changing on different timescales.

The size of this cohort warrants a note on interpretation. With 133,306 patients, differences of a few tenths of a percentage point reach conventional statistical significance, and several of our adjusted estimates are significant while being of negligible magnitude. The odds ratio of 1.03 for stage IV disease during the COVID-19 period corresponds to an absolute difference of 0.5 percentage points, or five additional patients per 1000, and the odds ratio of 1.06 for organ-specific metastasis corresponds to 1.4 percentage points. Neither would be perceptible in any individual clinic, and neither should be described as a substantial shift in presentation. By contrast, the increase in treatment delay during 2022–2023 corresponds to 4.3 percentage points, or 43 additional patients per 1000 waiting more than 90 days for treatment, an order of magnitude larger and of a scale that would be apparent at the service level. The same consideration applies to the early mortality finding: a hazard ratio of 1.05 implies an absolute difference bounded by five percent of the baseline 30-day mortality, which is necessarily well below one percentage point in a population whose 12-month mortality is approximately 35%. We have therefore reported absolute proportions alongside every relative estimate, and we interpret the delay findings as the substantive signal in these data while treating the stage, metastasis, and early-mortality findings as statistically detectable but clinically marginal.

The increase in metastatic presentation during the COVID-19 period is consistent with other observational evidence reporting reduced detection of earlier-stage lung cancer over the same interval. Kava et al. reported a 13.6% reduction in US lung cancer incidence between 2019 and 2020 and an increased proportion of distant-stage diagnoses [3]. Dyas et al. similarly identified 15% fewer newly diagnosed non-small-cell lung cancers in 2020, more advanced clinical TNM stage, and lower probabilities of treatment or primary resection [4]. Reduced low-dose computed tomography screening, avoidance of healthcare facilities, delayed evaluation of respiratory symptoms, and restricted access to invasive procedures are plausible explanations for these patterns, although our design cannot distinguish between them [19]. The absence of an increase in overall stage III–IV disease in our cohort, despite modest increases in stage IV disease and recorded metastases, suggests a shift concentrated at the metastatic end of the spectrum rather than across all stage categories.

The lower odds of advanced-stage and metastatic presentation during 2022–2023 may indicate partial restoration of diagnostic activity and evaluation of patients deferred earlier in the pandemic. However, national evidence through 2022 also found that a small increase in early-stage diagnoses coexisted with treatment initiation approximately nine days later than before the pandemic [3,7]. Improved stage distribution therefore does not necessarily indicate complete recovery of cancer care. Expanded screening eligibility, changes in low-dose computed tomography uptake, incidental detection through thoracic imaging, secular improvements in diagnostic pathways, and changes in registry case composition may also have contributed. Accordingly, the post-COVID-19 estimates should be interpreted as period associations rather than causal effects of healthcare-system recovery. The persistence of prolonged treatment delay into 2022–2023, at odds greater than during the acute pandemic period itself, indicates that the designation of this period as post-COVID-19 reflects chronology rather than restored system capacity.

Treatment patterns reinforced this divergence. Although pulmonary resection among patients with stage I–II disease was unchanged during the COVID-19 period, it was less frequent during 2022–2023, when sublobar resection also became more common relative to lobectomy. This pattern should not be attributed solely to the pandemic because evidence supporting lung-sparing surgery emerged during the study period. JCOG0802/WJOG4607L reported superior overall survival after segmentectomy in selected patients with peripheral clinical stage IA tumours measuring 2 cm or less, despite more local recurrence [20]. CALGB/Alliance 140503 subsequently demonstrated noninferior disease-free survival and similar overall survival with sublobar resection among patients with peripheral, node-negative stage IA non-small-cell lung cancer measuring no more than 2 cm [21]. The post-COVID-19 increase in sublobar resection is compatible with both pandemic-related pressures and an evidence-based secular change in thoracic surgical practice, and our design cannot apportion it between them.

Treatment initiation more than 90 days after diagnosis was 11% more likely during COVID-19 and 45% more likely after COVID-19 than before the pandemic. Earlier findings have been heterogeneous. A German cancer-centre study identified a longer interval from multidisciplinary review to resection, without clinically important delays in systemic therapy or radiotherapy [5], whereas another study reported an approximately 10-day increase from diagnosis to systemic treatment early in the pandemic followed by later recovery [7]. Persistent delays in our national cohort may reflect accumulated backlogs, workforce and procedural constraints, and increasingly complex molecular testing and treatment planning. Nevertheless, SEER records time to the first documented treatment rather than each component of the care pathway; longer intervals may partly represent more extensive staging or biomarker evaluation and should not automatically be considered avoidable delays.

Mortality associations varied with time since diagnosis. COVID-period diagnosis was associated with a small increase in 30-day all-cause mortality, but not at 90 or 180 days, and the estimated hazard was lower at 365 days. The early excess may reflect acute healthcare disruption, delayed presentation among unstable patients, peri-diagnostic COVID-19 morbidity, or residual differences in severity. The later estimates require considerably more caution and should not be read as evidence of a protective effect of the pandemic on cancer outcomes.

Three considerations bear on this. The first point is competing mortality. Our own estimates illustrate its effect directly: at 365 days, the cause-specific hazard ratio for lung cancer death was 0.91 (95% CI, 0.87–0.95), whereas the Fine-Gray subdistribution hazard ratio was 0.96 (95% CI, 0.94–0.99). The cause-specific model removes other-cause deaths by censoring and so assumes that such censoring is independent of cancer mortality, an assumption that is implausible when an excess competing cause acts preferentially on frail patients with advanced malignancy. The subdistribution model retains those deaths in the risk set, and the attenuation of the estimate toward the null is a measure of how much of the apparent cancer-specific reduction does not survive that accounting. The second point is cause-of-death ascertainment. A patient with lung cancer who dies with COVID-19 may be certified with COVID-19 as the underlying cause, which simultaneously removes the death from the cancer-specific numerator and adds it to the competing-risk category. National data show precisely this pattern, with cancer as an underlying cause of death continuing its previous decline during 2020 while deaths listing cancer as a contributing cause increased [22], and lung cancer mortality among older adults was lower than expected during parts of 2020, plausibly because COVID-19 acted as a competing cause [23]. Our cancer-specific analyses also excluded 787 patients with a missing or unknown cause of death; if that exclusion varied by diagnostic period, as pandemic-era certification practice makes plausible, the cancer-specific estimates would be biased by an amount we cannot quantify.

The third point is residual confounding and selection. Comorbidity burden is the principal determinant of competing non-cancer mortality and is not recorded in SEER, so the subdistribution estimates are particularly exposed to its absence; performance status, smoking history, and molecular status are likewise unavailable and all bear on 12-month survival. In addition, lung cancer incidence fell by approximately 14% during 2020 [3], which raises the possibility that some patients who would have been diagnosed and died rapidly never entered the registry at all. The COVID-era cohort would then be partly conditioned on having survived long enough to be diagnosed, which would depress observed later mortality without any change in the effectiveness of care. Treatment advances over calendar time, which affect all periods, may also contribute. Taken together, these considerations indicate that all-cause mortality is the more robust endpoint in this setting, because it is unaffected by cause-of-death misclassification, and that the cancer-specific and subdistribution estimates are best read as supporting analyses whose direction is informative but whose magnitude should not be interpreted quantitatively. We note also that a relative reduction of 4% with an upper confidence limit of 0.99 is a small effect that reaches significance largely because of the size of the cohort.

The persistence of treatment delay beyond the acute pandemic period has a practical consequence that the period-level analyses above cannot address. If patients continue to wait longer for treatment, clinicians must decide which patients to prioritise, and that decision is made for one patient at a time at the moment of diagnosis. This is the question the prediction analysis was designed to answer.

The prediction analysis was designed around the intended moment of use. Chemotherapy, radiotherapy, surgery type, reason for no surgery, and treatment delay occur after diagnosis and treatment decisions; including them would convert a presentation-time prediction into a retrospective description of a known treatment course. This follows the “no time machine” principle: predictors must be available when prediction is intended, and information generated after baseline can create label leakage [24]. The effect was substantial. In the 2022 cohort, the gradient-boosting ROC AUC decreased from 0.880 with post-diagnosis variables to 0.840 without them (difference, 0.041; 95% CI, 0.038–0.044), with comparable differences of 0.036–0.043 across the other algorithms (Table 5 and Table S6, and Figure 6). PR AUC decreased from 0.810 to 0.713, and the Brier score increased from 0.130 to 0.153. Moreover, “died before planned surgery” partly records the outcome itself, and treatment-delay missingness was strongly related to mortality. The post-diagnosis specification is therefore reported only as a labelled reference and should not be interpreted as a deployable model.

Under the primary diagnosis-only specification, all four algorithms performed similarly (Table 5 and Figure 3). Development out-of-fold ROC AUCs ranged from 0.829 to 0.833 and the temporal-validation values from 0.834 to 0.840, and precision–recall performance remained well above the prevalence baseline, which matters here because approximately one third of patients died within 12 months. The precision–recall analysis describes the proportion of true events among positive predictions [14]. Gradient boosting performed best according to the prespecified selection criterion (temporal-validation ROC AUC, 0.840; 95% CI, 0.834–0.845), but the margin over penalised logistic regression was 0.005 ROC AUC units with overlapping confidence intervals. And the inner cross-validated performance at the selected hyperparameters spanned only 0.004 across the four families. This consistency across model classes of very different flexibility indicates that the predictive information in these registry variables is close to additive, and that a penalised linear model recovers nearly all of it. Performance did not deteriorate in 2022 for any algorithm.

The paired comparison quantifies this directly. In the 2022 cohort, the difference in ROC AUC between gradient boosting and penalised logistic regression was 0.0046 (95% CI, 0.0035 to 0.0058; *p* < 0.001), and the two models ranked patients almost identically, with the predicted risks correlating at a Spearman coefficient of 0.988. The difference is therefore real and precisely estimated, and it is also negligible: with 22,012 participants and fully paired predictions, a difference of one two-hundredth of an area under the curve is detectable without being of any consequence for a decision made about a patient. This is a useful illustration of why statistical significance and clinical relevance must be assessed separately in studies of this size, and the same consideration applies to several of our epidemiological estimates.

Whether the margin matters clinically is answered by the decision-curve results. At a threshold probability of 0.30, the advantage of using the diagnosis-only model rather than treating all patients corresponds to approximately 138 additional true positives per 1000. The advantage of gradient boosting over penalised logistic regression at the same threshold corresponds to 1.3 per 1000 and did not exceed 3.6 per 1000 at any threshold between 0.10 and 0.50. At the locked operating point, the more complex model identified 74 more of the 7526 deaths with 11 fewer false positives. Essentially, the entire clinical gain therefore comes from using a model rather than a default strategy; the choice between these particular learners contributes on the order of one percent of it.

Calibration favoured the simpler model and is the stricter requirement, because a model may rank patients correctly while systematically misstating absolute risk [15,25]. On temporal validation, penalised logistic regression was the only one of the four whose calibration slope had a confidence interval containing unity (1.009; 95% CI, 0.980 to 1.038), whereas the intervals for gradient boosting, the support vector machine, and particularly random forest all excluded it. For the primary model, the slope of 1.031 (95% CI, 1.001 to 1.059) indicates a small compression of predicted risk toward the centre of the distribution; in the development cohort, the interval contained unity, so the compression emerged on temporal transfer rather than reflecting overfitting. In a cohort of 22,012 patients, departures of this magnitude are detectable without being clinically consequential, since a patient whose predicted risk is 80% has an observed risk of approximately 82%. Overall risk was neither systematically over- nor under-estimated in a validation year in which observed mortality had fallen slightly from the development period (34.2% versus 35.3%), and the predicted and observed mortality agreed to within 1.3 percentage points across deciles spanning observed risks from 1.9% to 81.8%. These findings nevertheless indicate that periodic recalibration, by intercept-and-slope updating on contemporaneous data, would be prudent if the model were deployed across successive years.

The costs of the more flexible model are not confined to performance metrics. A penalised regression model can be published as a complete equation with its coefficients, recalculated independently by anyone, and inspected directly as odds ratios without recourse to post hoc attribution methods; intercept-and-slope updating for a new setting is straightforward; and implementation requires no model-serving infrastructure. We therefore report gradient boosting as the primary model because it was chosen by a rule fixed in advance and changing that selection after inspecting the validation data would reintroduce precisely the optimism this study was designed to avoid, but we regard penalised logistic regression as a defensible and, in several respects, preferable choice for implementation.

On the balance of these considerations, we would expect a penalised logistic regression to be the better choice for implementation in most settings. It is the better-calibrated model on temporal validation, its difference in discrimination is below any plausible threshold of clinical relevance, it can be published in full as an equation and recalculated by hand or in a spreadsheet, its coefficients are directly inspectable as odds ratios, intercept-and-slope recalibration for a new setting is straightforward, and external validation by other groups requires no specialist infrastructure. We report gradient boosting as the primary model because it was selected by a rule fixed in advance, and revising that selection after inspecting the validation data would reintroduce the optimism this study was designed to avoid; but we would not argue for deploying the more complex model on the evidence presented here.

At the threshold of 0.341, selected using the Youden rule from development out-of-fold predictions and then locked, gradient boosting achieved 0.816 sensitivity, 0.712 specificity, 0.596 positive predictive value, and 0.882 negative predictive value in 2022 (Supplementary Table S5 and Supplementary Figure S1). These measures are prevalence- and threshold-dependent: approximately one in eight patients classified as lower risk still died within a year, while two in five classified as higher risk survived. The appropriate threshold therefore depends on the consequences of the proposed intervention, and here the asymmetry favours sensitivity, because a patient incorrectly identified as high risk receives an earlier supportive-care conversation and closer follow-up, whereas one incorrectly identified as lower risk may lose the opportunity for timely advance-care planning. A lower threshold would be defensible if the triggered intervention were less burdensome. Decision curve analysis addressed the same question formally by comparing model-guided action with intervening in all or no patients [16]: all diagnosis-only models provided positive net benefit across threshold probabilities from 0.01 to 0.80 and exceeded the treat-all strategy from approximately 0.10 upward, which became harmful beyond the outcome prevalence of 0.342 (Figure 5).

What a clinician gains from these estimates is best judged against the classification already in use. Stage performs reasonably well: 58.6% of patients with stage IV disease died within 12 months, compared with 7.0%, 13.7%, and 26.7% for stages I, II, and III. It is nonetheless coarse. Of the 7526 deaths in the 2022 cohort, 1525 occurred in patients with stage I–III disease, among whom 12-month mortality was 13.2% overall but was concentrated in subgroups identifiable at diagnosis by age, tumour size, grade, and histology; a further 325 occurred among the 743 patients whose stage was not recorded, a group with 43.7% mortality for whom stage-based triage provides no guidance but for whom the model still generates an estimate. Classifying all patients with stage IV disease as high risk would flag 44.0% of the cohort and correctly flag 5676 of those who died, whereas the model flagged 46.8% and correctly flagged 6143. Expressed per 1000 patients assessed, the model flags 28 more, of whom 21 die within the year and 7 do not, and leaves 21 fewer deaths unflagged, with a positive predictive value marginally higher rather than lower. This is a real improvement obtained at modest additional workload and without loss of precision, but it is an incremental refinement of existing practice rather than a categorical advance, and we would not present it otherwise.

The plausible applications are consequently those in which early identification changes management without requiring certainty. Early specialist palliative-care referral in advanced lung cancer has been associated with improved quality of life and mood [8], and the principal practical obstacle is identifying candidates promptly at diagnosis rather than late in the disease course. Similar considerations apply to prioritisation for expedited multidisciplinary review and to intensified follow-up during periods of constrained capacity, which our findings indicate have persisted beyond the acute pandemic period. Conversely, the model does not support withholding active treatment, does not estimate benefit from any specific therapy, and cannot replace clinical assessment of performance status and comorbidity, neither of which is captured in SEER. Its appropriate role is to raise a question earlier than it would otherwise be raised, not to answer it. For clinical interpretation of the decision curve, the potential intervention is an expedited multidisciplinary and supportive-care assessment rather than a model-directed change in anticancer treatment. Model-guided use would reserve this additional assessment for patients exceeding a locally selected risk threshold; the threshold would depend on available resources and would require external validation before implementation. The appropriate claim is correspondingly bounded. The model does not identify a group that stage-based assessment misses entirely; it reorders patients somewhat more finely, chiefly by recovering risk among patients with stage I–III disease and among those whose stage was not recorded. Whether an increment of this size justifies implementation depends on the cost of the triggered intervention and on local capacity and cannot be settled by the discrimination and net-benefit results reported here.

Replacing overall stage with separate T, N, and M categories changed discrimination by no more than approximately 0.002 ROC AUC units, despite nominal statistical significance for two algorithms in the large validation cohort (Supplementary Table S6). Calibration was also essentially unchanged. Stage group was therefore retained in the primary model for interpretability. The sensitivity analysis nevertheless clarified attribution: the M category became dominant when components were entered separately, demonstrating how correlated representations of disease extent can divide importance across variables.

SHAP values describe how each variable contributes to the model’s predictions and are not estimates of causal effects on mortality [26]. The contributions were clinically coherent in direction and stable between cohorts, which supports the model’s internal consistency but says nothing about mechanism. This distinction matters most for the reference specification: the prominence of chemotherapy, surgery type and treatment delay in that model reflects confounding by indication, because patients with better underlying prognosis are more likely to receive and complete treatment [27], and the attributions must not be read as treatment effects.

The 2022 cohort provides a genuine out-of-period evaluation, in the sense that it was never used for model fitting, hyperparameter selection, model selection, or threshold derivation, and that no recalibration was applied to it. It nevertheless constitutes a weak test of transportability, and the distinction matters for how these results should be read. Development and validation cohorts were drawn from the same registries, abstracted by the same registrars working from the same coding manuals, subject to the same case-ascertainment and quality-control procedures, and ascertained for vital status by the same mechanism. The only planned difference between them is the calendar period. A design of this kind establishes that the model remains stable as the population evolves over time within a fixed data-generating process. It cannot establish that the model would behave as reported in a population ascertained differently, coded differently, or treated within a different health system.

The components most likely to prove unstable are those tied to the United States context during this period. Stage distribution at presentation reflects national screening uptake, insurance coverage, and access to diagnostic services, and differs substantially between health systems. Baseline 12-month mortality of 34.2% is not a property of the model but of the population it was evaluated in, and the positive and negative predictive values reported here, together with the net-benefit calculations, depend directly upon it. Treatment-delay patterns reflect capacity and referral practice specific to this system and period. Experience with clinical prediction models suggests that discrimination tends to transport more readily than calibration, so a plausible pattern in a new setting would be a preserved ranking of patients alongside systematic displacement of absolute risk, which would compromise precisely the use we propose. A setting with a materially different outcome prevalence would require recalibration of the intercept before the predicted probabilities could be communicated to patients, even if the ordering of patients transferred intact.

An adequate external validation would require an independent cohort containing at least 100 to 200 events, which is the conventional minimum for stable estimation of calibration, and should report the calibration slope and intercept with CIs and a flexible calibration curve alongside discrimination, rather than discrimination alone. Decision-curve analysis should be repeated at thresholds relevant to the local care pathway, since net benefit depends on prevalence. Where calibration proves inadequate, intercept-and-slope updating on local data is the appropriate remedy and preserves the model structure. We have sought to make such studies practicable rather than merely to recommend them: the complete fitted pipeline and the full analysis code are publicly available, and penalised logistic regression, which performed equivalently to the more complex learners, can be published as a closed-form equation with its coefficients and evaluated independently without specialist infrastructure. Until an independent external validation has been performed, this model should be regarded as a research instrument and should not be used to inform individual patient care.

This study has limitations, the most consequential of which arise from the variables SEER does not collect. Eastern Cooperative Oncology Group performance status is unavailable, yet it is among the strongest determinants of both early mortality and eligibility for active treatment. Its absence has two distinct consequences. In the epidemiological analyses, the lower odds of pulmonary resection observed during 2022–2023 cannot be separated from a possible change in the fitness of patients presenting for surgical assessment, so these estimates should not be read as evidence of changed surgical decision-making alone. In the prediction analyses, performance status is information a clinician possesses at the bedside, but the model does not, so the incremental value demonstrated here relative to stage-based classification may differ from the incremental value relative to an experienced clinician’s overall assessment, which was not evaluated.

Comorbidity burden, smoking history and pack-years, and pulmonary function are likewise unrecorded. These influence competing mortality, treatment tolerance, and the choice between lobectomy and sublobar resection, and their absence is a plausible contributor to the increase in sublobar resection during 2022–2023 that we could not disentangle from the concurrent publication of trial evidence supporting lung-sparing surgery. Because competing non-cancer mortality is itself driven largely by comorbidity, the Fine-Gray estimates are particularly sensitive to this omission, and the modestly lower subdistribution hazard observed during the COVID-19 period should be interpreted accordingly rather than as evidence of improved cancer control.

Molecular and biomarker characteristics, including EGFR, ALK, ROS1, and PD-L1 status, are not captured. Over the study period, these increasingly determined the first-line systemic therapy and are associated with substantial differences in survival in advanced disease. The relevant heterogeneity extends well beyond the presence or absence of a targetable alteration at diagnosis. Within EGFR-mutant disease, for example, acquired resistance to osimertinib is a principal determinant of outcome, and molecular studies have identified transcriptional mediators of that resistance, such as the long non-coding RNA (Ribonucleic Acid) lnc-TMEM132D-AS1, which is upregulated in resistant lung adenocarcinoma cells and in the plasma of patients whose disease has become resistant [28]. Prognostically informative molecular variation is not confined to adenocarcinoma: analyses across tumour types, including lung squamous cell carcinoma, indicate that expression-level biomarkers can carry prognostic information independent of routinely recorded clinicopathological variables [29]. Two consequences follow for the present model. First, patients who are indistinguishable on every variable available to us may differ substantially in their actual twelve-month risk, so a ceiling on achievable discrimination is imposed by the data rather than by the algorithm, and the values reported here should be regarded as a lower bound for a model with access to molecular information. Second, because the distribution of targetable alterations and of access to the corresponding therapies differs between populations and health systems, this omission bears directly on transportability: a model fitted where a high proportion of patients receive targeted therapy may not transfer to a setting where testing or access differs, even if the registry variables are identically coded. This limitation compounds rather than overlaps with the restriction to first-course treatment, since SEER records neither the molecular basis for a treatment decision nor what followed it, so subsequent lines of therapy, treatment response, and progression are unknown.

Individual SARS-CoV-2 infection status and local pandemic intensity are not available. Period-based categorisation therefore acts as a proxy for exposure to healthcare disruption and cannot distinguish patients whose outcomes were affected by infection itself from those affected by delayed presentation or constrained capacity. The small excess in 30-day mortality during the COVID-19 period is consistent with either mechanism, and the data cannot adjudicate between them. Pandemic intensity also varied markedly across registries and over time, so a single national period effect averages heterogeneous local experiences.

Further limitations are methodological. Calendar-period comparisons are observational and may capture secular changes in screening, staging, systemic therapy, and surgery, in addition to pandemic effects. Chemotherapy and radiotherapy are coded as “No/unknown,” and time to first treatment cannot distinguish delays in diagnosis, staging, molecular testing, multidisciplinary evaluation, or scheduling, so longer intervals should not automatically be considered avoidable. Residual confounding is possible throughout and is of particular concern for the cancer-specific and competing-risk estimates for the reasons set out above. Changes in cause-of-death classification during the pandemic are documented nationally and cannot be corrected for in these data. We did not perform a quantitative bias analysis for cause-of-death misclassification, and the magnitude of the later cancer-specific estimates should be regarded as uncertain for this reason. Within the prediction analyses, missingness in some staging variables was outcome-related, and single median or modal imputation cannot represent informative missingness. The prespecified sensitivity analysis quantifies the consequence: retaining explicit missingness indicators improved temporal-validation discrimination in all four algorithms, by 0.0036 to 0.0048 ROC AUC units, with Brier scores falling by 0.0015 to 0.0020 and calibration unchanged. The reported primary results are therefore conservative with respect to this limitation rather than optimistic. We did not perform multiple imputation, which is designed for estimation under a missing-at-random assumption and is less well-suited to the present situation: the missingness here is strongly outcome-related, and it will also be present when the model is used, since a patient whose staging work-up was incomplete at diagnosis will present to the model with the same values absent. Where missingness is both informative and persistent at deployment, retaining it as information is generally preferable to imputing it away, and the sensitivity analysis indicates that doing so would modestly improve rather than degrade performance [30]. Facility identifiers were unavailable, so between-centre heterogeneity could not be assessed. The comparison with stage-based classification was added after peer review rather than prespecified and should be regarded as exploratory, although it used the same locked threshold and validation cohort as the primary analysis. Finally, race and ethnicity were unavailable in the extract, so fairness across racial and ethnic groups could not be examined; the constraints on external validation discussed above apply with equal force here, and an external validation including these data would be required to establish equitable performance [31].

## 5. Conclusions

In this population-based study, the COVID-19 period was associated with modest increases in metastatic presentation and prolonged treatment delay rather than a uniform shift toward advanced-stage lung adenocarcinoma. Although stage distribution improved during 2022–2023, treatment delays and changes in surgical practice persisted, indicating that diagnostic and treatment pathways recovered at different rates. The early increase in all-cause mortality was not sustained. Lower later cancer-specific and subdistribution estimates should not be read as evidence of better cancer outcomes during the pandemic: competing mortality, pandemic-era changes in cause-of-death certification, unmeasured comorbidity, and possible selection of the diagnosed cohort all act in the same direction, and the attenuation of the subdistribution estimate relative to the cause-specific estimate indicates that competing mortality alone accounts for part of the apparent difference.

Because prioritisation under continuing capacity constraints remains a practical problem, we examined whether patients at high risk of early death can be identified at the moment of diagnosis. A model restricted to diagnosis-time variables predicted 12-month mortality with a temporal-validation ROC AUC of 0.840 and retained calibration without recalibration, stratifying patients across a 43.9-fold gradient of observed mortality from 1.9% in the lowest predicted-risk decile to 81.8% in the highest. Clinically, it correctly flagged 467 more of the patients who died within 12 months than classification by stage IV disease alone, at the cost of flagging 150 additional patients who survived the year, and it provided greater net benefit than intervening in all patients or in none across the full range of plausible decision thresholds. The appropriate role of such a model is to prompt earlier consideration of supportive-care involvement, expedited multidisciplinary review, and intensified follow-up, not to determine treatment.

Chemotherapy, radiotherapy, surgery type, reason for no surgery, and treatment delay were excluded from this model because none are available when the prediction would be made, and because one category of reason for no surgery records death before planned surgery and therefore encodes the outcome. A reference model containing them reached a ROC AUC of 0.880 against 0.840 for the diagnosis-only model, a difference of 0.041 (95% CI, 0.038–0.044). That figure describes retrospective explanation of outcomes in patients whose treatment course is already known, not prediction at presentation, and should not be cited as the expected performance of a bedside tool.

Four algorithm families of markedly different flexibility performed within 0.006 ROC AUC units of one another, and the paired difference between gradient boosting and penalised logistic regression was 0.0046 (95% CI, 0.0035 to 0.0058), corresponding to between 1.3 and 3.6 additional true positives per 1000 patients against 138 per 1000 for using any model rather than a default strategy. Penalised logistic regression was also the only model whose temporal-validation calibration slope was statistically indistinguishable from unity. In this setting, the predictive information in routinely recorded registry variables appears to be close to additive, and a simpler, well-calibrated, fully reportable model is a reasonable and arguably preferable choice for implementation. The clinically important decision is whether to use a model at all rather than which learner to use. Validation here was temporal and internal to a single registry system, which establishes stability over time but not transportability to other populations or health systems. Independent external validation, reporting calibration as well as discrimination and including performance status, comorbidity, molecular characteristics, and subgroup fairness, is required before this model is used in clinical practice. The fitted model and analysis code are published to make such evaluation straightforward.

## Figures and Tables

**Table 1:** Inclusion and exclusion criteria and derivation of the final study cohort.

Stage	Criteria Applied	Records or Patients Remaining
1. Initial SEER extraction	Included: malignant primary tumour of the lung and bronchus (SEER Site Recode ICD-O-3/WHO 2008); malignant behaviour according to ICD-O-3; diagnosis between 1 January 2018 and 31 December 2023; age 18 years or older; eligible reporting source with sufficient identifiers for patient-level processing. Excluded at extraction: non-malignant tumours; primary sites outside the lung and bronchus; diagnosis outside 2018–2023; age under 18 years; cases reported only by autopsy; cases reported only by death certificate.	287,147 tumour records from 279,666 patients
2. Patient-level record selection	Excluded: additional eligible lung tumour records belonging to the same patient, retaining only the chronologically earliest eligible record (7481 records removed).	279,666 patients
3. Adenocarcinoma cohort definition	Included: histology meeting the prespecified expanded lung adenocarcinoma definition (ICD-O-3 codes listed in the Supplementary Methods) together with acceptable microscopic confirmation. Excluded: histologies not meeting the expanded adenocarcinoma definition, and potential adenocarcinoma records without acceptable microscopic confirmation (146,360 patients removed by these criteria in combination).	133,306 patients
Final analytic cohort	—	133,306 patients

Autopsy-only and death-certificate-only cases were removed during the SEER extraction and were not present in the downloaded analytic records; their number cannot be reconstructed from the final dataset and must not be added to the exclusion counts shown. The 1625 records meeting the SEER-defined adenocarcinoma histology but lacking acceptable microscopic confirmation are contained within the 146,360 exclusions at stage 3 and are not counted separately. The final cohort comprised 45,873 patients diagnosed during 2018–2019 (pre-COVID-19), 42,006 during 2020–2021 (COVID-19), and 45,427 during 2022–2023 (post-COVID-19); this distribution is also given in Supplementary Table S1.

**Table 2:** Selected demographic, tumour, and treatment characteristics across the COVID-19 diagnostic periods: pre-COVID-19 (2018–2019), COVID-19 (2020–2021), and post-COVID-19 (2022–2023).

Characteristic	Total *n* = 133,306	Pre-COVID 2018–2019 *n* = 45,873	COVID-19 2020–2021 *n* = 42,006	Post-COVID 2022–2023 *n* = 45,427	*p* Value
Age at diagnosis, mean (SD)	70.0 (10.3)	69.8 (10.4)	70.0 (10.3)	70.4 (10.2)	<0.001
Female	72,401 (54.3%)	24,718 (53.9%)	22,654 (53.9%)	25,029 (55.1%)	<0.001
Married/partnered	69,225 (51.9%)	23,465 (51.2%)	22,022 (52.4%)	23,738 (52.3%)	<0.001
Median household income ≥ $110,000	29,560 (22.2%)	9780 (21.3%)	9729 (23.2%)	10,051 (22.1%)	<0.001
Nonmetropolitan	16,414 (12.3%)	5651 (12.3%)	5254 (12.5%)	5509 (12.1%)	0.074
Stage IV	59,884 (44.9%)	20,975 (45.7%)	19,448 (46.3%)	19,461 (42.8%)	<0.001
Any organ-specific metastasis	58,548 (43.9%)	20,101 (43.8%)	19,073 (45.4%)	19,374 (42.6%)	<0.001
Surgery performed	39,544 (29.7%)	13,481 (29.4%)	12,245 (29.2%)	13,818 (30.4%)	<0.001
Pulmonary resection	39,194 (29.4%)	13,383 (29.2%)	12,120 (28.9%)	13,691 (30.1%)	<0.001
Sublobar resection	10,133 (7.6%)	3297 (7.2%)	3040 (7.2%)	3796 (8.4%)	<0.001
Radiotherapy, yes	48,113 (36.1%)	17,168 (37.4%)	15,188 (36.2%)	15,757 (34.7%)	<0.001
Chemotherapy, yes	52,905 (39.7%)	18,265 (39.8%)	16,772 (39.9%)	17,868 (39.3%)	0.160
Treatment delay > 90 days	12,927 (9.7%)	3811 (8.3%)	3796 (9.0%)	5320 (11.7%)	<0.001

Values are n (%) unless otherwise indicated. The *p* values refer to comparisons across the three diagnostic periods. Full demographic and clinicopathological distributions are provided in Supplementary Tables S3 and S4.

**Table 3:** Adjusted associations between the COVID-19 diagnostic period and clinical outcomes, with observed proportions and absolute differences, comparing COVID-19 (2020–2021) and post-COVID-19 (2022–2023) with pre-COVID-19 (2018–2019).

Outcome	Observed Proportion: Pre/COVID/Post	COVID-19 (2020–2021) vs. Pre-COVID-19 (2018–2019), OR (95% CI)	Absolute Difference (pp)	*p*-Value	Post-COVID-19 (2022–2023) vs. Pre-COVID-19 (2018–2019), OR (95% CI)	Absolute Difference (pp)	*p*-Value	N
Advanced stage (III–IV)	62.1/61.9/58.5%	1.00 (0.98–1.03)	−0.2	0.838	0.88 (0.85–0.90)	−3.6	<0.001	128,655
Stage IV	47.4/47.9/44.4%	1.03 (1.00–1.06)	+0.5	0.030	0.90 (0.88–0.93)	−3.0	<0.001	128,655
Any organ-specific metastasis	44.4/45.8/42.9%	1.06 (1.04–1.09)	+1.4	<0.001	0.95 (0.92–0.98)	−1.5	<0.001	131,957
Pulmonary resection in stage I–II	64.1/64.0/62.2%	0.99 (0.94–1.04)	−0.1	0.660	0.92 (0.88–0.96)	−1.9	0.001	50,376
Sublobar resection vs. lobectomy	—	1.02 (0.95–1.09)	—	0.647	1.20 (1.13–1.28)	—	<0.001	31,299
Treatment delay > 90 days	10.2/11.1/14.5%	1.11 (1.06–1.17)	+0.9	<0.001	1.45 (1.39–1.52)	+4.3	<0.001	106,563

**Table 4:** Twelve-month overall and cancer-specific mortality across the COVID-19 era, restricted to diagnoses during 2018–2022. Because patients diagnosed in 2023 lacked complete 12-month follow-up, the post-COVID-19 category in these analyses comprises 2022 only and is designated early post-COVID-19 (2022).

Outcome/Model	Comparison/Time Point	Measure	Adjusted Estimate (95% CI)	*p*-Value	N
12-month overall mortality: time-varying Cox model					
Overall mortality	COVID vs. Pre-COVID: Day 30	HR	1.05 (1.02–1.08)	0.002	106,466
Overall mortality	COVID vs. Pre-COVID: Day 90	HR	1.01 (0.98–1.03)	0.612	106,466
Overall mortality	COVID vs. Pre-COVID: Day 180	HR	0.98 (0.95–1.01)	0.144	106,466
Overall mortality	COVID vs. Pre-COVID: Day 365	HR	0.95 (0.92–0.99)	0.012	106,466
Overall mortality	Early post-COVID (2022) vs. Pre-COVID	HR	0.99 (0.96–1.02)	0.498	106,466
12-month cancer-specific mortality: time-varying Cox model					
Cancer-specific mortality	COVID vs. Pre-COVID: Day 30	HR	1.02 (0.99–1.06)	0.202	105,679
Cancer-specific mortality	COVID vs. Pre-COVID: Day 90	HR	0.97 (0.95–1.00)	0.032	105,679
Cancer-specific mortality	COVID vs. Pre-COVID: Day 180	HR	0.94 (0.91–0.97)	<0.001	105,679
Cancer-specific mortality	COVID vs. Pre-COVID: Day 365	HR	0.91 (0.87–0.95)	<0.001	105,679
Cancer-specific mortality	Early post-COVID (2022) vs. Pre-COVID	HR	0.96 (0.93–0.99)	0.011	105,679
12-month lung cancer mortality: Fine-Gray competing-risk model					
Lung cancer death	COVID vs. Pre-COVID	sHR	0.96 (0.94–0.99)	0.003	105,679
Lung cancer death	Early post-COVID (2022) vs. Pre-COVID	sHR	0.95 (0.92–0.98)	0.001	105,679

HRs were obtained from stratified Cox models using stage and marital status as stratification variables, with adjustment for age, sex, median household income, and rurality. Because the proportional-hazards assumption was violated for the COVID-period coefficient, COVID-versus-pre-COVID HRs are reported at prespecified follow-up times. The early post-COVID coefficient was modelled as time-invariant. Fine-Gray models treated other-cause death as the competing event and were adjusted for age, sex, marital status, median household income, rurality, and stage. The cancer-specific analyses include 105,679 patients against 106,466 in the all-cause analyses. The difference of 787 patients arises from exclusion of those with missing or unknown underlying cause of death, as specified in Section 2.7; if such exclusion varied by diagnostic period, the cancer-specific estimates would be biased by an amount that cannot be quantified from these data. CI, confidence interval; HR, hazard ratio; sHR, subdistribution hazard ratio.

**Table 5:** Discrimination and calibration of the diagnosis-only prediction models (Specification A) in the development and temporal validation cohorts.

Cohort	Model	ROC AUC (95% CI)	PR AUC (95% CI)	Outcome Prevalence	Brier Score	Scaled Brier	Calibration Intercept	Calibration Slope
Development (2018–2021, pooled out-of-fold)	Logistic Regression	0.830 (0.827–0.832)	0.703 (0.697–0.709)	0.353	0.1590	0.304	+0.000	1.000
	Random Forest	0.829 (0.826–0.832)	0.702 (0.696–0.708)	0.353	0.1596	0.301	+0.002	1.081
	Gradient Boosting	0.833 (0.830–0.836)	0.710 (0.705–0.716)	0.353	0.1574	0.311	+0.001	1.012
	Support Vector Machine	0.829 (0.826–0.832)	0.702 (0.697–0.709)	0.353	0.1594	0.302	+0.031	1.022
Temporal validation (2022)	Logistic Regression	0.835 (0.829–0.841)	0.703 (0.691–0.715)	0.342	0.1548	0.312	−0.022	1.009
	Random Forest	0.836 (0.830–0.842)	0.703 (0.693–0.716)	0.342	0.1547	0.312	−0.014	1.092
	Gradient Boosting	0.840 (0.834–0.845)	0.713 (0.702–0.725)	0.342	0.1527	0.321	−0.018	1.031
	Support Vector Machine	0.834 (0.828–0.840)	0.702 (0.689–0.713)	0.342	0.1552	0.310	+0.005	1.034

The prediction cohorts are defined by availability of 12-month follow-up rather than by the three-period diagnostic categorisation and are not interchangeable with it: the development cohort comprises diagnoses during 2018–2021 excluding 598 patients with insufficient follow-up and no observed death, and the temporal-validation cohort comprises diagnoses during 2022 under the same rule. Outcome prevalence is the proportion of patients who died within 12 months in the corresponding cohort and is the PR AUC that a non-informative model would achieve; the equivalent null value for ROC AUC is 0.500 regardless of prevalence. Because prevalence differed between the two cohorts (0.353 and 0.342), PR AUC values are not directly comparable across cohorts and should be read against the prevalence in the same row.

**Table 6:** Effect of predictor specification on performance in the 2022 temporal validation cohort. Specification C is a reference analysis only and cannot be used at initial presentation.

Specification	Model	ROC AUC (95% CI)	PR AUC (95% CI)	Brier Score	Scaled Brier	Calibration Slope	Δ ROC AUC vs. A
A—diagnosis only, stage group (primary)	Logistic Regression	0.835 (0.829–0.841)	0.703 (0.691–0.715)	0.1548	0.312	1.009	-
	Random Forest	0.836 (0.830–0.842)	0.703 (0.693–0.716)	0.1547	0.312	1.092	-
	Gradient Boosting	0.840 (0.834–0.845)	0.713 (0.702–0.725)	0.1527	0.321	1.031	-
	Support Vector Machine	0.834 (0.828–0.840)	0.702 (0.689–0.713)	0.1552	0.310	1.034	-
B—diagnosis only, T/N/M components (sensitivity)	Logistic Regression	0.834 (0.829–0.840)	0.703 (0.692–0.714)	0.1552	0.310	1.012	−0.0007
	Random Forest	0.838 (0.833–0.844)	0.708 (0.697–0.720)	0.1538	0.317	1.112	+0.0021
	Gradient Boosting	0.840 (0.835–0.846)	0.716 (0.705–0.727)	0.1522	0.324	1.035	+0.0010
	Support Vector Machine	0.834 (0.828–0.839)	0.702 (0.691–0.713)	0.1555	0.309	1.033	−0.0002
C—post-diagnosis variables included (reference only; not usable at presentation)	Logistic Regression	0.872 (0.867–0.877)	0.792 (0.783–0.801)	0.1348	0.401	1.017	+0.0367
	Random Forest	0.879 (0.874–0.884)	0.808 (0.800–0.816)	0.1312	0.417	1.194	+0.0430
	Gradient Boosting	0.880 (0.876–0.885)	0.810 (0.802–0.818)	0.1298	0.423	1.075	+0.0409
	Support Vector Machine	0.870 (0.865–0.875)	0.791 (0.782–0.800)	0.1352	0.399	1.038	+0.0357

Specification A (primary) contains only variables available at diagnosis and uses stage group; Specification B replaces stage group with the T, N and M components; Specification C additionally contains chemotherapy, radiotherapy, surgery type, reason for no surgery and time from diagnosis to first treatment. Specification C cannot be used at initial presentation and is reported only to quantify the optimism introduced by post-diagnosis information. The difference in ROC AUC relative to Specification A is shown in the final column; pairwise significance testing is reported in Supplementary Table S6. CIs are percentile bootstrap intervals from 500 resamples. ROC AUC, area under the receiver operating characteristic curve; PR AUC, area under the precision–recall curve. All estimates are from the 2022 temporal-validation cohort, in which the outcome prevalence was 0.342. A non-informative model would therefore achieve a PR AUC of 0.342 and a ROC AUC of 0.500.

## Data Availability

The data analysed in this study are publicly available from the National Cancer Institute’s SEER Program (https://seer.cancer.gov/data/, accessed on 11 August 2026). Access requires submission and approval of a SEER Research Data Agreement.

## Supplementary Materials

The following supporting information can be downloaded at https://www.mdpi.com/article/10.3390/diagnostics16203369/s1: Supplementary Table S1: Distribution of the final cohort by diagnostic period; Supplementary Table S2: Analysis-specific study populations; Supplementary Table S3: Complete baseline characteristics according to the COVID-19 diagnostic period; Supplementary Table S4: Complete tumour characteristics and treatment patterns according to the COVID-19 diagnostic period; Supplementary Table S5: Classification performance of the diagnosis-only models at the prespecified locked decision threshold; Supplementary Table S6: Pairwise comparison of predictor specifications in the 2022 temporal-validation cohort; Supplementary Table S7: Hyperparameter search space and selected values for every algorithm and specification; Supplementary Table S8: Agreement between predicted and observed 12-month mortality across deciles of predicted risk; Supplementary Table S9: STROBE statement checklist for the observational cohort component; Supplementary Table S10: TRIPOD + AI checklist for the prediction-model component; Supplementary Table S11: Sensitivity analysis of temporal-validation performance with and without explicit missingness indicators; Supplementary Figure S1: Confusion matrices for the diagnosis-only models in the development and 2022 temporal-validation cohorts; Supplementary Figure S2: Variable attribution with and without post-diagnosis information for gradient boosting; Supplementary Figure S3: Discrimination of the T/N/M sensitivity models (Specification B), development and 2022 cohorts; Supplementary Figure S4: Discrimination of the reference specification containing post-diagnosis variables (Specification C), development and 2022 cohorts; Supplementary Figure S5: Calibration of Specifications B and C in both cohorts; Supplementary Figure S6: Shapley additive explanations for the T/N/M sensitivity model (Specification B); Supplementary Figure S7: Shapley additive explanations for the reference specification (Specification C); Supplementary Figure S8: Confusion matrices for Specifications B and C at their locked thresholds; Supplementary Data S1: Analysis results workbook, including the run configuration record (software versions, configuration parameters, random seed, and execution timestamp), per-model performance estimates, calibration bins, decision-curve values, threshold-selection results, hyperparameter search results, and subgroup performance; Supplementary Software S1: Python source code and execution scripts for the reported cohort construction, epidemiologic models, machine learning models, calibration, decision curve and SHAP analyses are available at https://github.com/omerecinar/MDPI-Manuscript-AllInOneCode (accessed on 2 September 2026).

## Abbreviations

The following abbreviations are used in this manuscript:

ALK	Anaplastic lymphoma kinase
AUC	Area under the curve
CI	Confidence interval
COVID-19	Coronavirus disease 2019
EGFR	Epidermal growth factor receptor
EOD	Extent of disease
HR	Hazard ratio
ICD-O3	International Classification of Diseases for Oncology, third edition
OR	Odds ratio
PD-L1	Programmed death-ligand 1
PR	Precision recall
RNA	Ribonucleic acid
ROC	Receiver operating characteristic
ROS1	ROS proto-oncogene 1
SARS-CoV-2	Severe acute respiratory syndrome coronavirus 2
SD	Standard deviation
SEER	Surveillance, Epidemiology, and End Results
SHAP	SHapley additive exPlanations
sHR	Subdistribution hazard ratio
STROBE	Strengthening the Reporting of Observational Studies in Epidemiology
SVM	Support Vector Machine
TNM	Tumour, node, metastasis
TRIPOD + AI	Transparent Reporting of a multivariable prediction model for Individual Prognosis Or Diagnosis extended to artificial intelligence
