# Supplementary material for: Stage Migration, Treatment Patterns, and Machine Learning-Based Prediction of 12-Month Mortality in Lung Adenocarcinoma Across the COVID-19 Era: A SEER Population-Based Study
Source: Diagnostics (Basel). 2026 Oct 16;16(20):3369. doi: 10.3390/diagnostics16203369 (PMC13703649; doi:10.3390/diagnostics16203369)
Supplement: Supplementary file 1 [file diagnostics-16-03369-s001.zip › Supplementary Figures and Tables.pdf]

Supplementary Information

Cohort identification, eligibility criteria, exclusions, and analysis populations

Data source and initial case selection

Cases were identified from the SEER 17 Registries Research Plus database linked to time-dependent county attributes. The initial extraction was restricted to malignant primary tumours of the lung and bronchus diagnosed from 2018 through 2023.

Inclusion criteria

- Diagnosis between January 1, 2018 and December 31, 2023.
- Primary site classified as Lung and Bronchus in the SEER Site Recode ICD-O-3/WHO 2008.
- Malignant behaviour according to ICD-O-3.
- Age 18 years or older in the analytic cohort.
- Eligible reporting source and sufficient identifiers for patient-level processing.
- Histology consistent with the prespecified expanded lung adenocarcinoma definition and microscopic confirmation.

Exclusion criteria

- Cases reported only by autopsy.
- Cases reported only by death certificate.
- Diagnosis outside 2018-2023.
- Non-malignant tumours or tumours outside the lung and bronchus site definition.
- Patients younger than 18 years.
- Additional eligible lung tumour records belonging to the same patient after the earliest eligible record was retained.
- Histologies not meeting the expanded adenocarcinoma definition.
- Potential adenocarcinoma records without acceptable microscopic confirmation.

Important counting note. Autopsy-only and death-certificate-only cases were excluded during the SEER extraction and were not present in the downloaded analytic records. Their numbers therefore cannot be reconstructed from the final dataset and must not be added to the later exclusion counts.

Patient-level record selection

The merged adult lung cancer file contained 287,147 eligible tumour records representing 279,666 unique patients. Patient ID and record number uniquely identified tumour records. Patients were ordered by patient ID, diagnosis year, and numeric record number. For patients with more than one eligible lung tumour record, only the chronologically earliest eligible lung record was retained. This removed 7,481 additional tumour records and produced a patient-level dataset of 279,666 unique individuals.

The retained record is described as the patient's earliest eligible lung cancer record. It should not be described as the patient's first-ever malignant primary because the selection was performed within the eligible lung cancer records available for this study.

Adenocarcinoma cohort definition

ICD-O-3 histology codes were converted to numeric form and classified using the prespecified expanded lung adenocarcinoma definition. Acceptable diagnostic confirmation included positive histology, positive exfoliative cytology, positive microscopic confirmation with the method not specified, or positive histology supported by immunophenotyping and/or genetic studies. Clinical diagnosis alone, radiographic diagnosis without microscopic confirmation, laboratory-marker-only diagnosis, unknown confirmation, and other nonmicroscopic categories were not considered acceptable confirmation for the adenocarcinoma cohort.

Among 279,666 unique adult patients with an eligible lung cancer record, 146,360 did not meet the combined expanded adenocarcinoma histology and diagnostic-confirmation definition. The final adenocarcinoma cohort therefore contained 133,306 patients. The 1,625 potential SEER-defined adenocarcinoma records removed because microscopic confirmation was

absent are included within the 146,360 combined exclusions and must not be counted a second time. The derivation of the cohort is shown as Figure 1 of the main manuscript, and the inclusion and exclusion criteria applied at each stage are summarized in main-manuscript Table 1.

COVID-19 diagnostic periods

Supplementary Table S1. Distribution of the final cohort by diagnostic period

**Diagnostic period**	**Diagnosis years**	**Patients, *n***
Pre-COVID	2018-2019	45,873
COVID	2020-2021	42,006
Post-COVID	2022-2023	45,427
Total	2018-2023	133,306

Analysis-specific populations and exclusions

Supplementary Table S2. Analysis-specific study populations

Analysis	Analysis-specific restriction or exclusion	Analysed *n*
Advanced stage (III-IV)	Unknown stage excluded (4,551); missing model covariates excluded (100)	128,655
Stage IV	Unknown stage excluded (4,551); missing model covariates excluded (100)	128,655
Any organ-specific metastasis	Indeterminate metastatic status or missing model covariates excluded (1,349)	131,957
Pulmonary resection in stage I-II	Restricted to stage I-II (50,457); missing outcome or covariates excluded (81)	50,376
Sublobar resection vs lobectomy	Restricted to stage I-II patients undergoing either procedure with complete covariates and tumor size	31,299
Treatment delay >90 days	Unknown treatment timing (25,129) or unknown stage (4,551) excluded; 2,937 met both criteria	106,563
12-month all-cause survival	2023 diagnoses excluded for incomplete follow-up; indeterminate outcomes excluded	Varied by model
C 12-month cancer-specific survival	As above, with unknown underlying cause of death additionally excluded (787)	Varied by model
Model development	Diagnoses 2018-2021; follow-up under 12 months without observed death excluded (598)	87,281
Temporal validation	Diagnoses 2022; 2023 diagnoses excluded for incomplete follow-up; follow-up under 12 months without observed death excluded	22,012

Final analytic cohort

The primary epidemiologic analyses included 133,306 unique adults with microscopically confirmed lung adenocarcinoma diagnosed during 2018-2023. The full cohort was used for descriptive stage, metastasis, and treatment comparisons, with missing categories shown explicitly. Complete-case restrictions were applied only within the corresponding adjusted models. Survival analyses used diagnosis years with sufficient follow-up and therefore did not treat the exclusion of 2023 diagnoses as an exclusion from the main epidemiologic cohort.

Supplementary Tables and Figures

This file contains detailed descriptive distributions, classification metrics, pairwise model comparisons, hyperparameter specifications, and supporting model-interpretation figures referenced in the main manuscript.

Supplementary Table S3. Complete baseline characteristics according to the COVID-19 diagnostic period

Characteristic	Total *n*=133,306	Pre-COVID 2018-2019 *n*=45,873	COVID 2020-2021 *n*=42,006	Post-COVID 2022-2023 *n*=45,427	p-value
**Age at diagnosis, mean (SD)**	70.0 (10.3)	69.8 (10.4)	70.0 (10.3)	70.4 (10.2)	<0.001
**Age group, years**					**<0.001**
**18-49**	4,082 (3.1%)	1,435 (3.1%)	1,291 (3.1%)	1,356 (3.0%)	
**50-59**	15,752 (11.8%)	5,992 (13.1%)	5,047 (12.0%)	4,713 (10.4%)	
**60-69**	41,946 (31.5%)	14,350 (31.3%)	13,134 (31.3%)	14,462 (31.8%)	
**70-79**	46,709 (35.0%)	15,710 (34.2%)	14,825 (35.3%)	16,174 (35.6%)	
**>=80**	24,817 (18.6%)	8,386 (18.3%)	7,709 (18.4%)	8,722 (19.2%)	
**Sex**					**<0.001**
**Female**	72,401 (54.3%)	24,718 (53.9%)	22,654 (53.9%)	25,029 (55.1%)	
**Male**	60,905 (45.7%)	21,155 (46.1%)	19,352 (46.1%)	20,398 (44.9%)	
**Marital status**					**<0.001**
**Married/partnered**	69,225 (51.9%)	23,465 (51.2%)	22,022 (52.4%)	23,738 (52.3%)	
**Single**	21,563 (16.2%)	7,157 (15.6%)	6,763 (16.1%)	7,643 (16.8%)	
**Divorced/separated**	17,136 (12.9%)	5,992 (13.1%)	5,449 (13.0%)	5,695 (12.5%)	
**Widowed**	20,815 (15.6%)	7,194 (15.7%)	6,501 (15.5%)	7,120 (15.7%)	
**Unknown**	4,567 (3.4%)	2,065 (4.5%)	1,271 (3.0%)	1,231 (2.7%)	
**Median household income**					**<0.001**
**<$70,000**	28,031 (21.0%)	9,863 (21.5%)	8,537 (20.3%)	9,631 (21.2%)	
**$70,000-$89,999**	38,339 (28.8%)	16,977 (37.0%)	11,439 (27.2%)	9,923 (21.8%)	
**$90,000-$109,999**	37,375 (28.0%)	9,253 (20.2%)	12,300 (29.3%)	15,822 (34.8%)	
**>=$110,000**	29,560 (22.2%)	9,780 (21.3%)	9,729 (23.2%)	10,051 (22.1%)	
**Missing**	1 (0.0%)	0	1 (0.0%)	0	
**Rurality**					**0.074**
**Large metropolitan**	80,396 (60.3%)	27,536 (60.0%)	25,469 (60.6%)	27,391 (60.3%)	
**Small/medium metropolitan**	36,392 (27.3%)	12,652 (27.6%)	11,245 (26.8%)	12,495 (27.5%)	
**Nonmetropolitan**	16,414 (12.3%)	5,651 (12.3%)	5,254 (12.5%)	5,509 (12.1%)	
**Missing**	104 (0.1%)	34 (0.1%)	38 (0.1%)	32 (0.1%)	
**Primary tumour site**					**<0.001**
**Main bronchus**	2,476 (1.9%)	850 (1.9%)	775 (1.8%)	851 (1.9%)	
**Upper lobe**	70,313 (52.7%)	24,053 (52.4%)	22,144 (52.7%)	24,116 (53.1%)	
**Middle lobe**	6,172 (4.6%)	2,096 (4.6%)	1,922 (4.6%)	2,154 (4.7%)	
**Lower lobe**	38,890 (29.2%)	13,446 (29.3%)	12,174 (29.0%)	13,270 (29.2%)	
**Overlapping lung lesion**	1,168 (0.9%)	360 (0.8%)	402 (1.0%)	406 (0.9%)	
**Lung, NOS**	14,287 (10.7%)	5,068 (11.0%)	4,589 (10.9%)	4,630 (10.2%)	
**Laterality**					**<0.001**
**Right**	76,072 (57.1%)	26,155 (57.0%)	23,967 (57.1%)	25,950 (57.1%)	
**Left**	51,780 (38.8%)	17,835 (38.9%)	16,255 (38.7%)	17,690 (38.9%)	
**Bilateral, single primary**	1,523 (1.1%)	467 (1.0%)	440 (1.0%)	616 (1.4%)	
**Paired site, laterality unknown**	3,753 (2.8%)	1,345 (2.9%)	1,282 (3.1%)	1,126 (2.5%)	
**Other/unspecified**	178 (0.1%)	71 (0.2%)	62 (0.1%)	45 (0.1%)	
**Derived summary grade**					**<0.001**
**Grade category 1**	11,125 (8.3%)	4,005 (8.7%)	3,491 (8.3%)	3,629 (8.0%)	
**Grade category 2**	26,446 (19.8%)	9,203 (20.1%)	8,196 (19.5%)	9,047 (19.9%)	
**Grade category 3**	23,326 (17.5%)	8,557 (18.7%)	7,247 (17.3%)	7,522 (16.6%)	
**Grade category 4**	170 (0.1%)	74 (0.2%)	38 (0.1%)	58 (0.1%)	
**High grade**	1 (0.0%)	0	0	1 (0.0%)	
**Unknown/not assessed**	72,238 (54.2%)	24,034 (52.4%)	23,034 (54.8%)	25,170 (55.4%)	

**Diagnostic confirmation**					**<0.001**
**Positive histology**	120,309 (90.3%)	40,975 (89.3%)	37,883 (90.2%)	41,451 (91.2%)	
**Positive exfoliative cytology**	12,971 (9.7%)	4,886 (10.7%)	4,118 (9.8%)	3,967 (8.7%)	
**Other microscopic confirmation**	26 (0.0%)	12 (0.0%)	5 (0.0%)	9 (0.0%)	

Supplementary Table S4. Complete tumour characteristics and treatment patterns according to the COVID-19 diagnostic period

Characteristic	Total *n*=133,306	Pre-COVID 2018-2019 *n*=45,873	COVID 2020-2021 *n*=42,006	Post-COVID 2022-2023 *n*=45,427	p-value
**TUMOR CHARACTERISTICS**					
**AJCC EOD 2018 stage**					**<0.001**
**Stage I**	40,644 (30.5%)	13,432 (29.3%)	12,448 (29.6%)	14,764 (32.5%)	
**Stage II**	9,813 (7.4%)	3,349 (7.3%)	3,020 (7.2%)	3,444 (7.6%)	
**Stage III**	18,414 (13.8%)	6,529 (14.2%)	5,701 (13.6%)	6,184 (13.6%)	
**Stage IV**	59,884 (44.9%)	20,975 (45.7%)	19,448 (46.3%)	19,461 (42.8%)	
**Unknown**	4,551 (3.4%)	1,588 (3.5%)	1,389 (3.3%)	1,574 (3.5%)	
**Stage availability**					**0.340**
**Known stage**	128,755 (96.6%)	44,285 (96.5%)	40,617 (96.7%)	43,853 (96.5%)	
**Unknown stage**	4,551 (3.4%)	1,588 (3.5%)	1,389 (3.3%)	1,574 (3.5%)	
**Broad stage**					**<0.001**
**Early stage (I-II)**	50,457 (37.9%)	16,781 (36.6%)	15,468 (36.8%)	18,208 (40.1%)	
**Advanced stage (III-IV)**	78,298 (58.7%)	27,504 (60.0%)	25,149 (59.9%)	25,645 (56.5%)	
**Unknown**	4,551 (3.4%)	1,588 (3.5%)	1,389 (3.3%)	1,574 (3.5%)	
**Tumour size**					**<0.001**
**<=20 mm**	36,348 (27.3%)	12,106 (26.4%)	11,268 (26.8%)	12,974 (28.6%)	
**21-30 mm**	24,793 (18.6%)	8,612 (18.8%)	7,608 (18.1%)	8,573 (18.9%)	
**31-50 mm**	28,890 (21.7%)	10,097 (22.0%)	9,020 (21.5%)	9,773 (21.5%)	
**51-70 mm**	13,797 (10.3%)	4,691 (10.2%)	4,562 (10.9%)	4,544 (10.0%)	
**>70 mm**	9,550 (7.2%)	3,169 (6.9%)	3,188 (7.6%)	3,193 (7.0%)	
**Missing**	19,928 (14.9%)	7,198 (15.7%)	6,360 (15.1%)	6,370 (14.0%)	
**Metastasis at diagnosis - Yes**					
**Bone**	26,316 (19.7%)	9,035 (19.7%)	8,575 (20.4%)	8,706 (19.2%)	<0.001
**Brain**	18,210 (13.7%)	6,234 (13.6%)	5,998 (14.3%)	5,978 (13.2%)	<0.001
**Liver**	9,908 (7.4%)	3,410 (7.4%)	3,162 (7.5%)	3,336 (7.3%)	<0.001
**Lung**	17,793 (13.3%)	6,273 (13.7%)	5,753 (13.7%)	5,767 (12.7%)	<0.001
**Distant lymph node**	9,808 (7.4%)	3,351 (7.3%)	3,117 (7.4%)	3,340 (7.4%)	<0.001
**Other**	26,827 (20.1%)	8,888 (19.4%)	8,836 (21.0%)	9,103 (20.0%)	<0.001
**Any organ-specific metastasis**	58,548 (43.9%)	20,101 (43.8%)	19,073 (45.4%)	19,374 (42.6%)	<0.001
**Separate ipsilateral tumour nodules**					**<0.001**
**No separate nodules**	91,795 (68.9%)	31,739 (69.2%)	28,641 (68.2%)	31,415 (69.2%)	
**Same lobe**	7,100 (5.3%)	2,567 (5.6%)	2,227 (5.3%)	2,306 (5.1%)	
**Different ipsilateral lobe**	5,022 (3.8%)	1,819 (4.0%)	1,560 (3.7%)	1,643 (3.6%)	
**Same and different lobes**	5,023 (3.8%)	1,737 (3.8%)	1,631 (3.9%)	1,655 (3.6%)	
**Ipsilateral lobe unspecified**	8,915 (6.7%)	3,330 (7.3%)	2,851 (6.8%)	2,734 (6.0%)	
**Missing/not assessed**	15,451 (11.6%)	4,681 (10.2%)	5,096 (12.1%)	5,674 (12.5%)	
**TREATMENT PATTERNS**					
**Primary-site surgery**					**<0.001**
**No surgery**	93,418 (70.1%)	32,308 (70.4%)	29,665 (70.6%)	31,445 (69.2%)	
**Surgery performed**	39,544 (29.7%)	13,481 (29.4%)	12,245 (29.2%)	13,818 (30.4%)	
**Missing**	344 (0.3%)	84 (0.2%)	96 (0.2%)	164 (0.4%)	
**Type of primary-site surgery**					**<0.001**
**Local tumour destruction**	350 (0.3%)	98 (0.2%)	125 (0.3%)	127 (0.3%)	
**Sublobar resection**	10,133 (7.6%)	3,297 (7.2%)	3,040 (7.2%)	3,796 (8.4%)	

**Lobectomy**	27,891 (20.9%)	9,585 (20.9%)	8,758 (20.8%)	9,548 (21.0%)	
**Extended lobectomy**	633 (0.5%)	261 (0.6%)	167 (0.4%)	205 (0.5%)	
**Pneumonectomy**	337 (0.3%)	159 (0.3%)	99 (0.2%)	79 (0.2%)	
**Extended/radical pneumonectomy**	16 (0.0%)	8 (0.0%)	4 (0.0%)	4 (0.0%)	
**Resection/surgery, NOS**	184 (0.1%)	73 (0.2%)	52 (0.1%)	59 (0.1%)	
**Missing**	344 (0.3%)	84 (0.2%)	96 (0.2%)	164 (0.4%)	
**Pulmonary resection**					**<0.001**
**No pulmonary resection**	93,768 (70.3%)	32,406 (70.6%)	29,790 (70.9%)	31,572 (69.5%)	
**Pulmonary resection**	39,194 (29.4%)	13,383 (29.2%)	12,120 (28.9%)	13,691 (30.1%)	
**Missing**	344 (0.3%)	84 (0.2%)	96 (0.2%)	164 (0.4%)	
**Radiotherapy**					**<0.001**
**No/unknown**	85,193 (63.9%)	28,705 (62.6%)	26,818 (63.8%)	29,670 (65.3%)	
**Yes**	48,113 (36.1%)	17,168 (37.4%)	15,188 (36.2%)	15,757 (34.7%)	
**Chemotherapy**					**0.160**
**No/unknown**	80,401 (60.3%)	27,608 (60.2%)	25,234 (60.1%)	27,559 (60.7%)	
**Yes**	52,905 (39.7%)	18,265 (39.8%)	16,772 (39.9%)	17,868 (39.3%)	
**Time from diagnosis to treatment**					**<0.001**
**Same day**	13,166 (9.9%)	5,468 (11.9%)	4,312 (10.3%)	3,386 (7.5%)	
**1-30 days**	31,319 (23.5%)	11,987 (26.1%)	10,284 (24.5%)	9,048 (19.9%)	
**31-60 days**	34,903 (26.2%)	11,473 (25.0%)	10,906 (26.0%)	12,524 (27.6%)	
**61-90 days**	15,862 (11.9%)	4,763 (10.4%)	4,796 (11.4%)	6,303 (13.9%)	
**>90 days**	12,927 (9.7%)	3,811 (8.3%)	3,796 (9.0%)	5,320 (11.7%)	
**Missing**	25,129 (18.9%)	8,371 (18.2%)	7,912 (18.8%)	8,846 (19.5%)	

Supplementary Table S5. Classification performance of the diagnosis-only models at the prespecified locked decision threshold

Cohort	Model	Thresh.	Confusion matrix [TN, FP, FN, TP]	Sens.	Spec.	PPV	NPV	F1	MCC
**Development (2018-2021, pooled out-of-fold)**	Logistic Regression	0.343	[39493, 16966, 5729, 25093]	0.814	0.700	0.597	0.873	0.689	0.491
	Random Forest	0.368	[39388, 17071, 5870, 24952]	0.810	0.698	0.594	0.870	0.685	0.485
	Gradient Boosting	0.341	[39503, 16956, 5639, 25183]	0.817	0.700	0.598	0.875	0.690	0.494
	Support Vector Machine	0.326	[39914, 16545, 6005, 24817]	0.805	0.707	0.600	0.869	0.688	0.490
**Temporal validation (2022)**	Logistic Regression	0.343	[10309, 4177, 1457, 6069]	0.806	0.712	0.592	0.876	0.683	0.493
	Random Forest	0.368	[10366, 4120, 1469, 6057]	0.805	0.716	0.595	0.876	0.684	0.495
	Gradient Boosting	0.341	[10320, 4166, 1383, 6143]	0.816	0.712	0.596	0.882	0.689	0.503
	Support Vector Machine	0.326	[10417, 4069, 1516, 6010]	0.799	0.719	0.596	0.873	0.683	0.493

The threshold was selected by the Youden rule applied to the pooled development out-of-fold predictions, then locked and applied unchanged to the 2022 cohort; no threshold optimisation was performed on the validation data. Five alternative threshold rules and their operating characteristics are reported in the supplementary workbook. TN, true negatives (survival correctly predicted); FP, false positives (survival incorrectly predicted as death); FN, false negatives (death incorrectly predicted as survival); TP, true positives (death correctly predicted); Sens., sensitivity for death; Spec., specificity for survival; PPV, positive predictive value for death; NPV, negative predictive value for survival; MCC, Matthews correlation coefficient. Positive and negative predictive values depend on outcome prevalence, which was 0.353 in the development cohort and 0.342 in the temporal-validation cohort, and would differ in a population of different outcome frequency. Sensitivity and specificity are prevalence-independent.

Supplementary Table S6. Pairwise comparison of predictor specifications in the 2022 temporal-validation cohort

Model	Comparison	ROC AUC, first	ROC AUC, second	Difference	95% CI of difference	P value
**Logistic Regression**	C vs A	0.8716	0.8349	+0.0367	0.0335 to 0.0399	8.6×10^{-112}
	C vs B	0.8716	0.8342	+0.0374	0.0341 to 0.0406	1.0×10^{-113}
	A vs B	0.8349	0.8342	+0.0007	-0.0007 to 0.0021	.353
**Random Forest**	C vs A	0.8791	0.8361	+0.0430	0.0397 to 0.0463	9.7×10^{-141}
	C vs B	0.8791	0.8382	+0.0409	0.0376 to 0.0442	1.2×10^{-130}
	A vs B	0.8361	0.8382	-0.0021	-0.0031 to -0.0011	5.3×10^{-5}
**Gradient Boosting**	C vs A	0.8805	0.8395	+0.0409	0.0378 to 0.0441	1.6×10^{-143}
	C vs B	0.8805	0.8405	+0.0400	0.0368 to 0.0431	7.7×10^{-138}
	A vs B	0.8395	0.8405	-0.0010	-0.0019 to -0.0000	.046
**Support Vector Machine**	C vs A	0.8697	0.8340	+0.0357	0.0324 to 0.0391	2.7×10^{-98}
	C vs B	0.8697	0.8338	+0.0359	0.0326 to 0.0393	1.1×10^{-99}
	A vs B	0.8340	0.8338	+0.0002	-0.0011 to 0.0015	.749

All specifications were evaluated in the same 2022 participants (n = 22,012), so the areas under the curve are correlated and the paired DeLong test is appropriate. In each row the difference is the first area minus the second. A, diagnosis-only with stage group; B, diagnosis-only with T, N and M components; C, reference specification containing post-diagnosis treatment variables. Every comparison of C against a diagnosis-only specification yielded a difference of 0.036 to 0.043 with P values below 10^{-97}. Comparisons of A against B yielded absolute differences of 0.0021 or less; two reached nominal significance in a sample of this size, but the confidence intervals exclude any difference exceeding 0.003, which is not clinically meaningful.

Supplementary Table S7. Hyperparameter search space and selected values for every algorithm and specification

Specification	Model	Search space (pre-specified)	Selected values	Inner CV ROC AUC
**A - diagnosis-only, stage group (primary)**	Logistic Regression	C: 0.01, 0.1, 1.0, 10.0	C = 0.1	0.8283
	Random Forest	n_estimators: 300, 600; max_depth: 8, 14, none; min_samples_leaf: 5, 20	n_estimators = 300; max_depth = none; min_samples_leaf = 5	0.8263
	Gradient Boosting	n_estimators: 200, 400; learning_rate: 0.05, 0.1; max_depth: 2, 3; subsample: 0.8	n_estimators = 400; learning_rate = 0.05; max_depth = 3; subsample = 0.8	0.8301
	Support Vector Machine	C: 0.01, 0.1, 1.0	C = 0.01	0.8276
**B - diagnosis-only, T/N/M components (sensitivity)**	Logistic Regression	C: 0.01, 0.1, 1.0, 10.0	C = 0.1	0.8278
	Random Forest	n_estimators: 300, 600; max_depth: 8, 14, none; min_samples_leaf: 5, 20	n_estimators = 600; max_depth = none; min_samples_leaf = 5	0.8278
	Gradient Boosting	n_estimators: 200, 400; learning_rate: 0.05, 0.1; max_depth: 2, 3; subsample: 0.8	n_estimators = 400; learning_rate = 0.05; max_depth = 3; subsample = 0.8	0.8318
	Support Vector Machine	C: 0.01, 0.1, 1.0	C = 0.01	0.8277
**C - post-diagnosis variables included (reference)**	Logistic Regression	C: 0.01, 0.1, 1.0, 10.0	C = 1.0	0.8663
	Random Forest	n_estimators: 300, 600; max_depth: 8, 14, none; min_samples_leaf: 5, 20	n_estimators = 600; max_depth = 14; min_samples_leaf = 5	0.8691
	Gradient Boosting	n_estimators: 200, 400; learning_rate: 0.05, 0.1; max_depth: 2, 3; subsample: 0.8	n_estimators = 400; learning_rate = 0.05; max_depth = 3; subsample = 0.8	0.8720
	Support Vector Machine	C: 0.01, 0.1, 1.0	C = 0.1	0.8648

Hyperparameters were selected by exhaustive grid search with three-fold stratified inner cross-validation, using the area under the receiver operating characteristic curve as the objective, on a stratified random subsample of 30,000 development-cohort patients. The final column is the inner cross-validated area under the curve at the selected configuration and is a tuning diagnostic, not a performance estimate; the internally validated and temporally validated estimates are given in main-manuscript Tables 4 and 5. The random seed was fixed at 42 for all model fitting and resampling. For the support vector machine, C refers to the inverse regularisation strength of the underlying linear model, whose decision scores were converted to probabilities by Platt scaling within a three-fold cross-validated wrapper. No other settings were tuned; all remaining parameters were left at their scikit-learn defaults.

Supplementary Table S8. Agreement between predicted and observed 12-month mortality across deciles of predicted risk for the primary diagnosis-only gradient-boosting model (Specification A)

Decile	*n*	Deaths	Mean predicted risk	Observed mortality (95% CI)	Difference
**Development cohort (2018-2021, pooled out-of-fold)**					
1	8,729	190	0.030	0.022 (0.019-0.025)	+0.009
2	8,728	431	0.056	0.049 (0.045-0.054)	+0.006
3	8,728	792	0.091	0.091 (0.085-0.097)	0.000
4	8,728	1,430	0.154	0.164 (0.156-0.172)	-0.010
5	8,728	2,299	0.260	0.263 (0.254-0.273)	-0.003
6	8,728	3,354	0.378	0.384 (0.374-0.395)	-0.006
7	8,728	4,190	0.485	0.480 (0.470-0.491)	+0.005
8	8,728	5,125	0.584	0.587 (0.577-0.598)	-0.003
9	8,728	5,946	0.683	0.681 (0.671-0.691)	+0.002
10	8,728	7,065	0.809	0.810 (0.801-0.818)	0.000
**Temporal-validation cohort (2022)**					
1	2,202	41	0.028	0.019 (0.014-0.025)	+0.010
2	2,201	95	0.052	0.043 (0.035-0.053)	+0.009
3	2,201	192	0.085	0.087 (0.076-0.100)	-0.002
4	2,201	312	0.142	0.142 (0.128-0.157)	+0.001
5	2,201	533	0.243	0.242 (0.225-0.261)	+0.001
6	2,201	781	0.362	0.355 (0.335-0.375)	+0.008
7	2,201	1,015	0.475	0.461 (0.440-0.482)	+0.013
8	2,201	1,269	0.575	0.577 (0.556-0.597)	-0.001
9	2,201	1,487	0.678	0.676 (0.656-0.695)	+0.003
10	2,202	1,801	0.807	0.818 (0.801-0.833)	-0.011

Patients were ranked by predicted probability of death within 12 months and divided into ten equal-sized groups. Development-cohort values are pooled out-of-fold predictions from five-fold stratified cross-validation; temporal-validation values were obtained by applying the model fitted on the whole development cohort to the 2022 cohort without refitting, recalibration, or threshold re-estimation. Observed mortality is the proportion of patients in each decile who died within 12 months, with 95% Wilson confidence intervals. Difference is mean predicted risk minus observed mortality, with positive values indicating over-prediction. In the temporal-validation cohort the maximum absolute difference was 0.013 and the mean predicted value fell within the 95% confidence interval of the observed proportion in nine of ten deciles, the exception being the lowest decile. In the development cohort the maximum absolute difference was smaller (0.010) but, because each decile contained approximately four times as many patients, the confidence intervals were correspondingly narrower and the predicted value fell outside the interval in three deciles; this reflects greater precision rather than poorer agreement. The pattern of slight over-prediction in the lower deciles and slight under-prediction in the highest decile of the 2022 cohort corresponds to the calibration slope of 1.031 reported in main-manuscript Table 4. Corresponding calibration curves are shown in main-manuscript Figure 4.

Reporting standards

This study reports two methodologically distinct components and is therefore assessed against two reporting guidelines. The observational cohort component, comprising the descriptive, regression, survival, and competing-risk analyses of stage, treatment, and mortality across the COVID-19 era, is reported in accordance with the STROBE statement for cohort studies. The prediction-model component, comprising the development and temporal validation of models for 12-month all-cause mortality, is reported in accordance with the TRIPOD + AI statement. Supplementary Tables S9 and S10 record where each item of the respective checklist is addressed.

The checklist topics below are abbreviated descriptions written for this study rather than the official wording, which is available from the STROBE Initiative (https://www.strobe-statement.org). Items marked "Not applicable" are identified as such with the reason. Page numbers refer to the submitted manuscript; "S-file" indicates that the item is addressed in this Supplementary Information rather than in the main text, and "n/a" indicates an item that does not apply, with the reason given in the preceding column.

Supplementary Table S9. STROBE statement: checklist of items addressed for the observational cohort component.

Item	Checklist topic	Where addressed in the manuscript	Page
1a	Study design indicated in the title or abstract	Title; Abstract, Methods	1
1b	Informative, balanced summary in the abstract	Abstract (structured: Background/Objectives, Methods, Results, Conclusions)	1
2	Scientific background and rationale	Section 1, paragraphs 1-3	2
3	Specific objectives and prespecified hypotheses	Section 1, final paragraph	3
4	Key elements of study design	Section 2.1	3
5	Setting, locations, and relevant dates of recruitment, exposure, follow-up, and data collection	Sections 2.1, 2.2, 2.3	3-6
6a	Eligibility criteria, sources, and methods of participant selection; methods of follow-up	Section 2.2; Table 1; Figure 1; Section 2.7	4-6, 8
6b	For matched studies, matching criteria and number of exposed and unexposed	Not applicable: the study is unmatched and uses calendar-period groups	n/a
7	Clearly defined outcomes, exposures, predictors, potential confounders, and effect modifiers	Sections 2.3-2.7; Section 2.9.2	6-9
8	Sources of data and details of methods of assessment for each variable	Sections 2.1, 2.2, 2.4-2.7; Supplementary Methods (ICD-O-3 definitions)	3-8
9	Efforts to address potential sources of bias	Sections 2.2 (case ascertainment and record selection), 2.8 (adjustment, competing risks), 2.9.2 (predictor timing); Section 4 (limitations)	4, 8-9, 28
10	How study size was arrived at	Section 2.2; Table 1; Figure 1; Section 2.9.1	4-6, 9
11	How quantitative variables were handled and why groupings were chosen	Sections 2.3-2.6, 2.8, 2.9.4	6-8, 10
12a	Statistical methods, including those used to control for confounding	Section 2.8	8
12b	Methods used to examine subgroups and interactions	Section 2.8; Section 3.3 (stage-stratified surgical analyses)	8, 13
12c	How missing data were addressed	Sections 2.2, 2.8, 2.9.4; Supplementary Table S2	5, 8, 10
12d	How loss to follow-up was addressed	Sections 2.7, 2.9.1 (exclusion of records with follow-up under 12 months and no observed death)	8-9
12e	Sensitivity analyses	Sections 2.8, 2.9.3; Section 3.8; Supplementary Table S6	8, 10, 21
13a	Numbers of individuals at each stage of	Section 2.2; Table 1; Figure 1; Supplementary	4-6

	the study	Tables S1-S2	
13b	Reasons for non-participation at each stage	Section 2.2; Table 1; Figure 1	4-6
13c	Flow diagram	Figure 1	6
14a	Characteristics of study participants and information on exposures and potential confounders	Section 3.1; Table 2; Supplementary Tables S3-S4	12-13
14b	Number of participants with missing data for each variable of interest	Supplementary Tables S3-S4 (missing categories displayed explicitly); Supplementary Table S2	S-file
14c	Summarised follow-up time	Sections 2.7, 3.4	8, 15
15	Numbers of outcome events or summary measures over time	Section 3.4; Table 4; Section 3.5	15-16
16a	Unadjusted and confounder-adjusted estimates with confidence intervals, and which confounders were adjusted for and why	Sections 3.3, 3.4; Tables 3 and 4	13-16
16b	Category boundaries when continuous variables were categorised	Sections 2.3-2.6	6-8
16c	Translation of relative risk into absolute risk for a meaningful time period	Sections 3.1, 3.2, 3.4; Section 3.7 (absolute numbers per 1,000 patients)	12-15, 20-21
17	Other analyses, including subgroup, interaction, and sensitivity analyses	Sections 3.5-3.9; Supplementary Tables S5-S8; Supplementary Figures S1-S8	16-23
18	Key results with reference to study objectives	Section 4, paragraph 1	23
19	Limitations, including sources of potential bias or imprecision and their direction and magnitude	Section 4, limitations paragraphs	28-29
20	Cautious overall interpretation considering objectives, limitations, multiplicity, and other evidence	Section 4; Section 5	23-30
21	Generalisability of the results	Section 4 (temporal validation and the limits of what it establishes); Section 5	27-30
22	Source of funding and role of funders	Funding statement	30

STROBE, Strengthening the Reporting of Observational Studies in Epidemiology. The checklist applies to the observational cohort component of this study. The prediction-model component is assessed separately in Supplementary Table S10.

Supplementary Table S10. TRIPOD + AI checklist: items addressed for the prediction-model component.

Item	Checklist topic	Where addressed in the manuscript	Page
1	Title identifies the study as developing or evaluating a prediction model, the target population, and the outcome	Title; see note below	1
2	Abstract: structured summary following the TRIPOD + AI for Abstracts checklist	Abstract	1
3a	Background: rationale, including reference to existing models	Section 1, paragraphs 1-4	2-3
3b	Intended use of the model in the clinical pathway and intended users	Section 2.9 (opening paragraph); Section 4 (plausible applications)	9, 27
4	Objectives, including whether the study describes development, evaluation, or both	Section 1, final paragraph; Sections 2.9, 2.9.3	3, 9-10
5a	Data source, including setting and eligibility	Sections 2.1, 2.2, 2.9.1	3-5, 9
5b	Dates of data collection and follow-up	Sections 2.1, 2.2, 2.9.1	3-5, 9
5c	Whether data were collected for a purpose other than this study	Section 2.1 (population-based registry surveillance data)	3
6a	Eligibility criteria for participants	Section 2.2; Table 1; Figure 1; Section 2.9.1	4-6, 9
6b	Details of treatments received, if relevant	Sections 2.6, 3.2; Supplementary Table S4	7-8, 13
6c	How treatments received were handled during model development and evaluation	Sections 2.9.2, 2.9.3; Section 3.8; Section 4 (post-diagnosis variables excluded from the primary model and retained only in a labelled reference specification)	9-10, 21, 25
7	Data preparation, including cleaning, harmonisation, and transformations	Section 2.9.4	10
8a	Outcome definition and how it was determined	Sections 2.7, 2.9.1	8-9
8b	Whether outcome assessors were blinded to predictor information	Not applicable: the outcome is all-cause death ascertained from registry follow-up and involves no assessor judgement	n/a
9a	Definition of all predictors and when they were measured	Sections 2.4-2.6, 2.9.2; Table 1	5-9
9b	Whether predictor assessors were blinded to outcome and other predictors	Not applicable: predictors are registry-coded fields abstracted independently of the present analysis	n/a
10	Sample size and how it was determined	Sections 2.2, 2.9.1; Table 1; Figure 1	4-6, 9
11	How missing data were handled	Section 2.9.4; Section 4 (informative missingness and direction of bias)	10, 29
12a	How the data were used (development, tuning, evaluation)	Section 2.9.5 (five-fold cross-validation with pooled out-of-fold predictions; temporal hold-out)	10-11
12b	How predictors were handled in the analysis	Section 2.9.4	10
12c	Model type, all model-building steps, and hyperparameter tuning	Section 2.9.5; Supplementary Table S7	10-11, S-file
12d	Heterogeneity in model performance across clusters	Not assessed: facility identifiers are unavailable in the extract; stated in Section 4 (limitations)	29
12e	Measures and plots used to evaluate performance, and their rationale	Section 2.9.7; Sections 3.5-3.7; Figures 3-5	11, 16-21
12f	Model updating or recalibration, if performed	Section 2.9.5 (none applied); Section 4 (recalibration pathway discussed)	11, 26
12g	How predictions were calculated	Section 2.9.9; analysis code repository	12
13	Approach to class imbalance, if any	Section 2.9.5 (no resampling applied; rationale	11

		given)	
14	Fairness: how performance was examined across relevant subgroups	Section 4 (limitations): race and ethnicity were unavailable in the extract, so fairness along that axis could not be assessed; see note below	29
15	Model output and, if thresholds were used, how they were derived	Sections 2.9.6, 3.6; Supplementary Table S5	11, 19-20
16	Differences between development and evaluation data	Sections 2.3, 2.9.1, 2.9.5; Section 4	6, 9, 11, 27-28
17	Ethical approval or exemption	Section 2.10; Institutional Review Board Statement	12, 30
18a	Funding and role of funders	Funding statement	30
18b	Conflicts of interest	Conflicts of Interest statement	31
18c	Availability of the study protocol	Not registered; the analysis plan is fully specified in Section 2.9 and in the published analysis code	9-12
18d	Registration	Not registered; stated here	n/a
18e	Data availability	Data Availability Statement	31
18f	Code availability	Supplementary Materials (Software S1); public code repository	30
19	Patient and public involvement	See note below	31
20a	Participant flow, including the number with and without the outcome	Section 2.2; Table 1; Figure 1; Sections 2.9.1, 3.5	4-6, 9, 16
20b	Participant characteristics, including predictor distributions	Section 3.1; Table 2; Supplementary Tables S3-S4	12-13
20c	Comparison of development and evaluation data distributions	Sections 2.9.1, 3.5; Supplementary Tables S1-S2	9, 16
21	Number of participants and outcome events in each analysis	Section 2.9.1; Supplementary Table S2; Tables 5-6	9, 17, 21
22	Model specification sufficient for others to generate predictions	Section 2.9.9; exported fitted pipeline and logistic regression coefficients in the code repository	12, 30
23a	Model performance with confidence intervals	Sections 3.5-3.7; Tables 5-6; Supplementary Tables S5, S8	16-21
23b	If applicable, performance across subgroups	See item 14 and the note below	29
24	Results of any model updating	None performed; stated in Section 2.9.5 and Section 4	11, 26
25	Interpretation of results in the context of objectives and other evidence	Section 4	23-29
26	Limitations, including those specific to the data and the model	Section 4, limitations paragraphs	28-29
27a	How the model could be used in practice and by whom	Section 2.9 (intended use); Section 4 (plausible applications)	9, 27
27b	How poor-quality or missing data should be handled at the point of use	Section 2.9.4; Section 4	10, 29
27c	Further research needed before clinical use	Section 4 (external validation requirements); Section 5	28-30

TRIPOD + AI, Transparent Reporting of a multivariable prediction model for Individual Prognosis Or Diagnosis, extended to artificial intelligence. The checklist applies to the prediction-model component of this study, developed and temporally validated as described in Sections 2.9 and 3.5-3.9. Items designated "development and evaluation" apply, since the model was developed in one calendar period and evaluated in another.

Supplementary Table S11. Sensitivity analysis: temporal-validation performance of the primary specification with and without explicit missingness indicators.

Model	Handling of missing data	ROC AUC	PR AUC	Brier score	Scaled Brier	Calibration intercept	Calibration slope
**Logistic Regression**	Imputation only (primary)	0.835	0.703	0.1548	0.312	-0.022	1.009
	Missingness indicators	0.840	0.710	0.1527	0.321	-0.022	1.017
**Random Forest**	Imputation only (primary)	0.836	0.703	0.1547	0.312	-0.014	1.092
	Missingness indicators	0.840	0.709	0.1530	0.320	-0.015	1.106
**Gradient Boosting**	Imputation only (primary)	0.840	0.713	0.1527	0.321	-0.019	1.031
	Missingness indicators	0.843	0.717	0.1512	0.328	-0.020	1.035
**Support Vector Machine**	Imputation only (primary)	0.834	0.702	0.1552	0.310	+0.005	1.034
	Missingness indicators	0.839	0.709	0.1533	0.319	+0.009	1.040

All estimates are from the 2022 temporal-validation cohort (n = 22,012; 7,526 deaths; outcome prevalence 0.342). The two configurations differ only in the handling of missing predictor values; hyperparameters, cross-validation partitions, the random seed, and the locked threshold rule were identical. The indicator configuration adds a binary missingness indicator alongside the median-imputed value for each continuous predictor and an explicit "missing" level for each categorical predictor, expanding the design matrix from 91 to 104 columns. Retaining missingness as information improved discrimination in all four algorithms, by 0.0036 to 0.0048 ROC AUC units, and lowered the Brier score by 0.0015 to 0.0020, while calibration was essentially unchanged; the primary results are therefore conservative rather than optimistic with respect to this limitation. PR AUC is prevalence-dependent, with a non-informative value equal to the outcome prevalence of 0.342. ROC AUC, area under the receiver operating characteristic curve; PR AUC, area under the precision-recall curve.

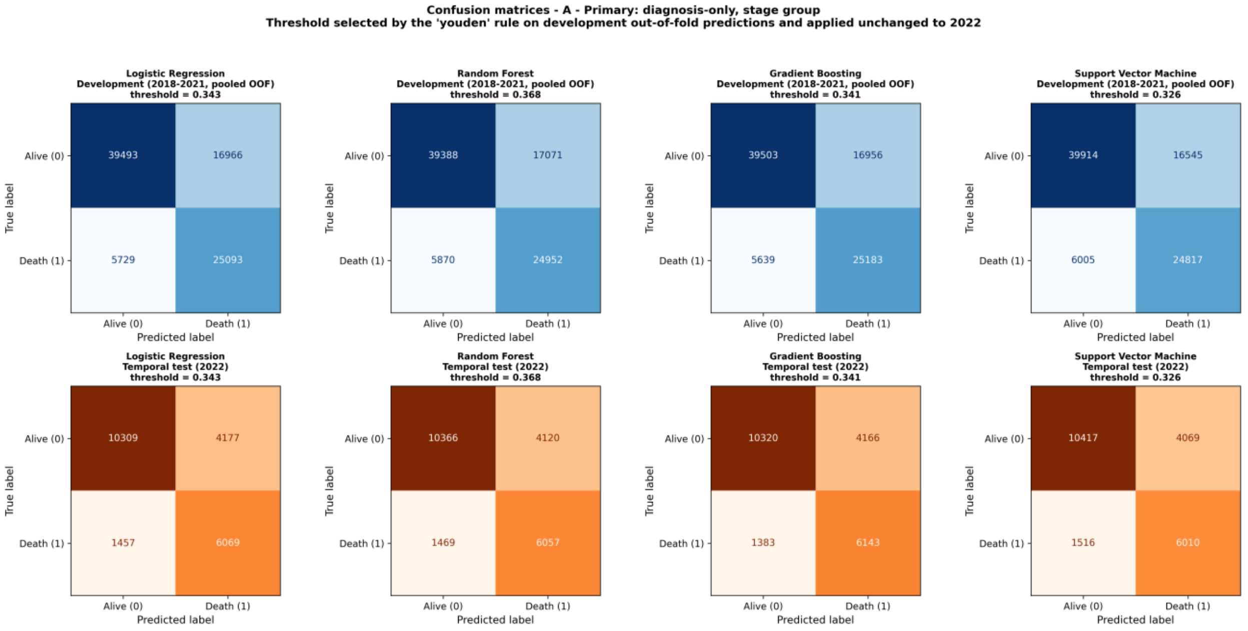

Supplementary Figure S1. Confusion matrices for the diagnosis-only models in the development and 2022 temporal-validation cohorts. The decision threshold shown above each matrix was selected by the Youden rule on development out-of-fold predictions and applied unchanged to the 2022 cohort.

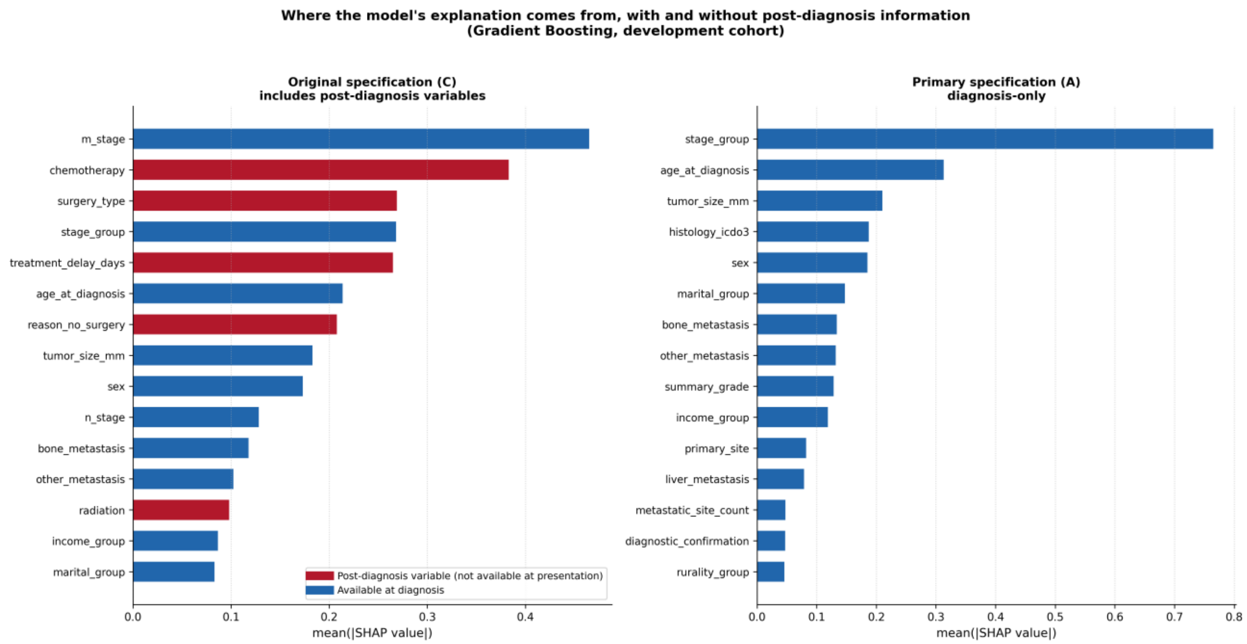

Supplementary Figure S2. Variable attribution with and without post-diagnosis information for gradient boosting. Post-diagnosis variables are shown in red in the reference specification.

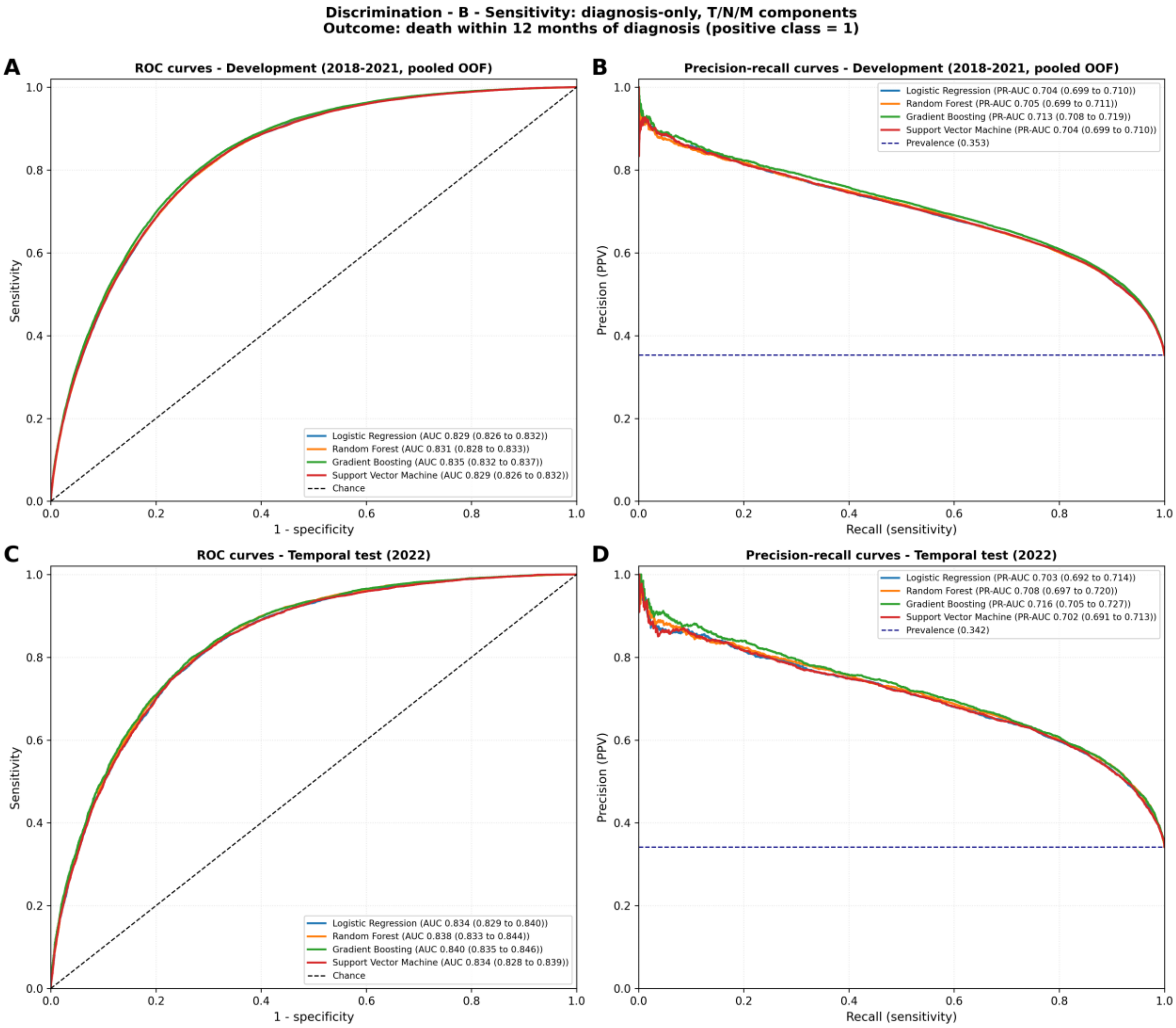

Supplementary Figure S3. Discrimination of the T/N/M sensitivity models (Specification B), development and 2022 cohorts. (A) Receiver operating characteristic curves, development cohort (pooled out-of-fold predictions); (B) precision-recall curves, development cohort; (C) receiver operating characteristic curves, 2022 temporal validation cohort; (D) precision-recall curves, 2022 temporal validation cohort. Areas under the curve are shown with percentile bootstrap 95% confidence intervals from 500 resamples. Dashed lines denote chance performance in the receiver operating characteristic panels and the outcome prevalence in the precision-recall panels.

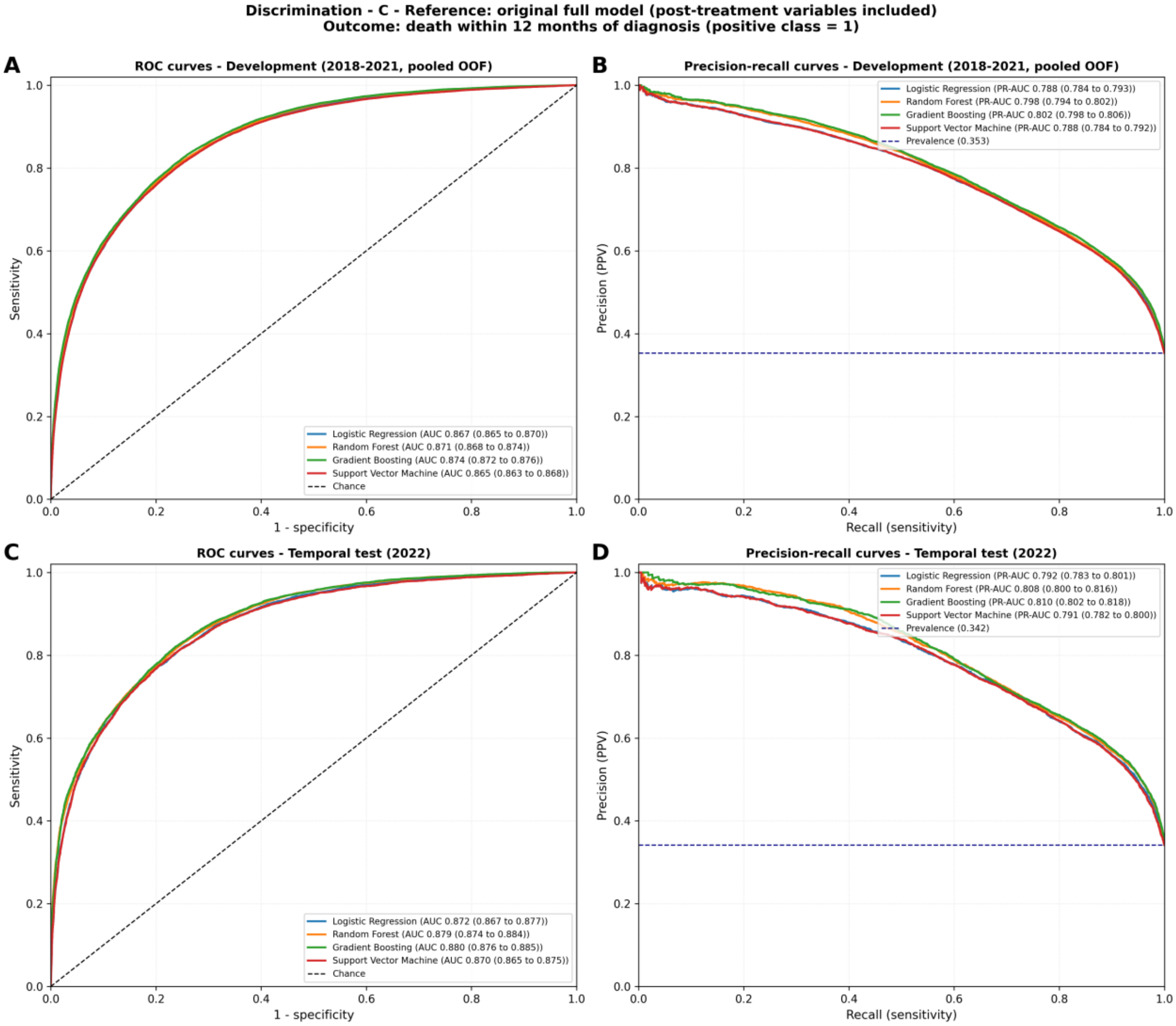

Supplementary Figure S4. Discrimination of the reference specification containing post-diagnosis variables (Specification C), development and 2022 cohorts. Panels are arranged as in Supplementary Figure S4. The higher areas under the curve shown here relative to Specifications A and B are attributable to chemotherapy, radiotherapy, surgery type, reason for no surgery and treatment delay, none of which is available at initial presentation; one category of reason for no surgery records death before planned surgery and therefore encodes the outcome directly. These values must not be interpreted as the performance attainable at the point of care. These values are reported as a reference analysis and are not attainable at initial presentation.

(A) Specification B - diagnosis-only, T/N/M components

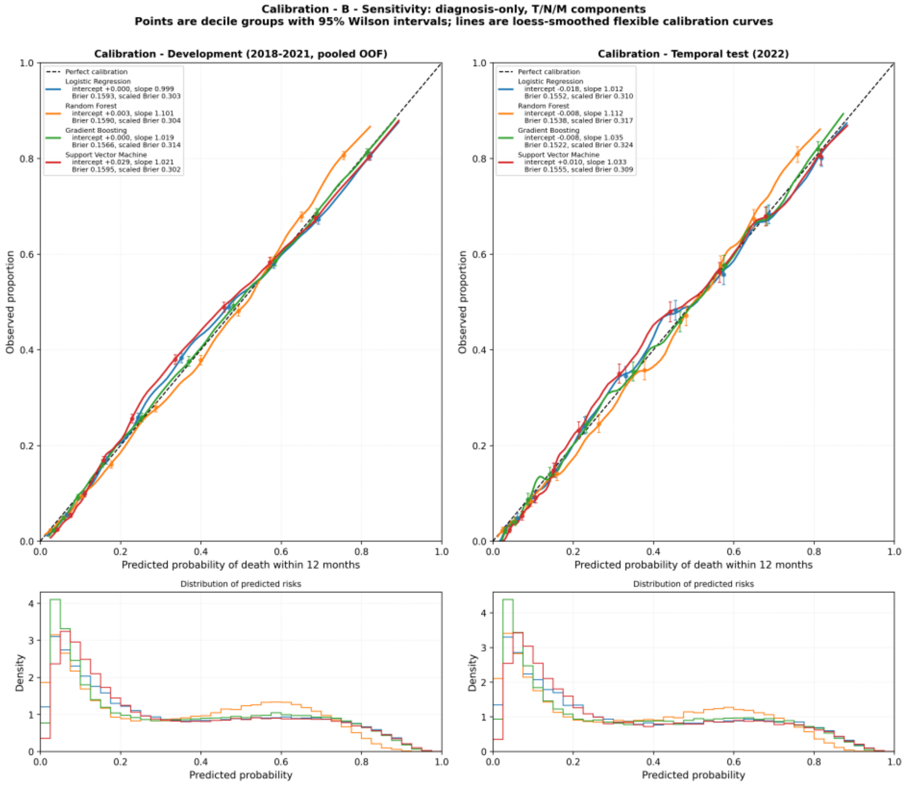

(B) Specification C - post-diagnosis variables included

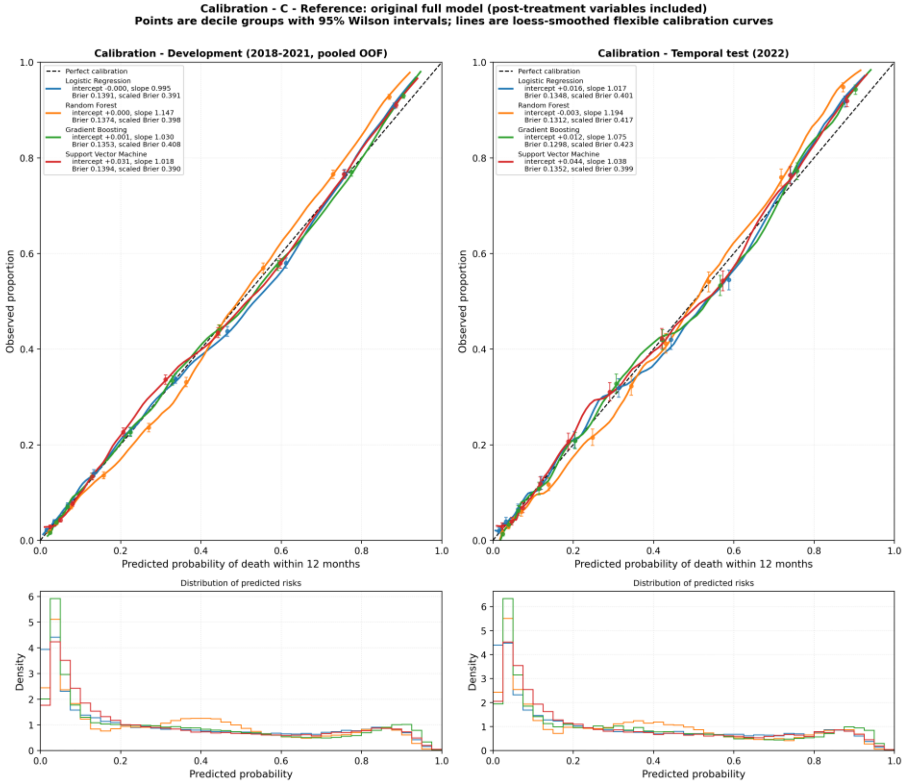

Supplementary Figure S5. Calibration of Specifications B and C in both cohorts. In each panel, the left plot is the development cohort and the right plot is the 2022 temporal validation cohort. Points are decile groups of predicted risk with 95% Wilson intervals, lines are loess-smoothed flexible calibration curves, and the dashed diagonal denotes perfect

calibration. The calibration intercept, calibration slope, Brier score and scaled Brier score for each model are given in the plot legends, and the lower sub-panels show the distribution of predicted probabilities. No recalibration was applied to the 2022 cohort in either specification.

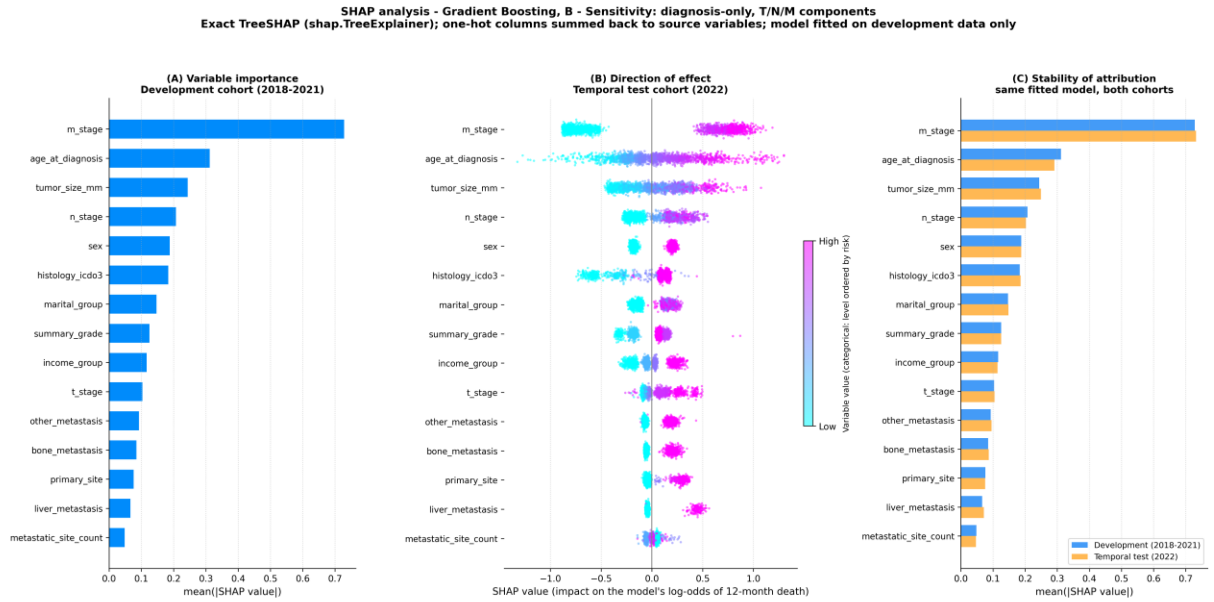

Supplementary Figure S6. Shapley additive explanations for the T/N/M sensitivity model (gradient boosting, Specification B). (A) Mean absolute Shapley value per variable in the development cohort. (B) Direction and magnitude of individual attributions in the 2022 temporal validation cohort; each point is one patient, coloured by the value of the variable, with categorical levels ordered by observed risk. (C) Comparison of mean absolute Shapley values between the two cohorts using the same fitted model. Attributions were computed by exact TreeSHAP from a model fitted on development data only, and indicator columns were summed back to their source variable before importance was computed. When the components are entered separately, M category becomes the dominant single variable, whereas in Specification A the same information is concentrated in stage group.

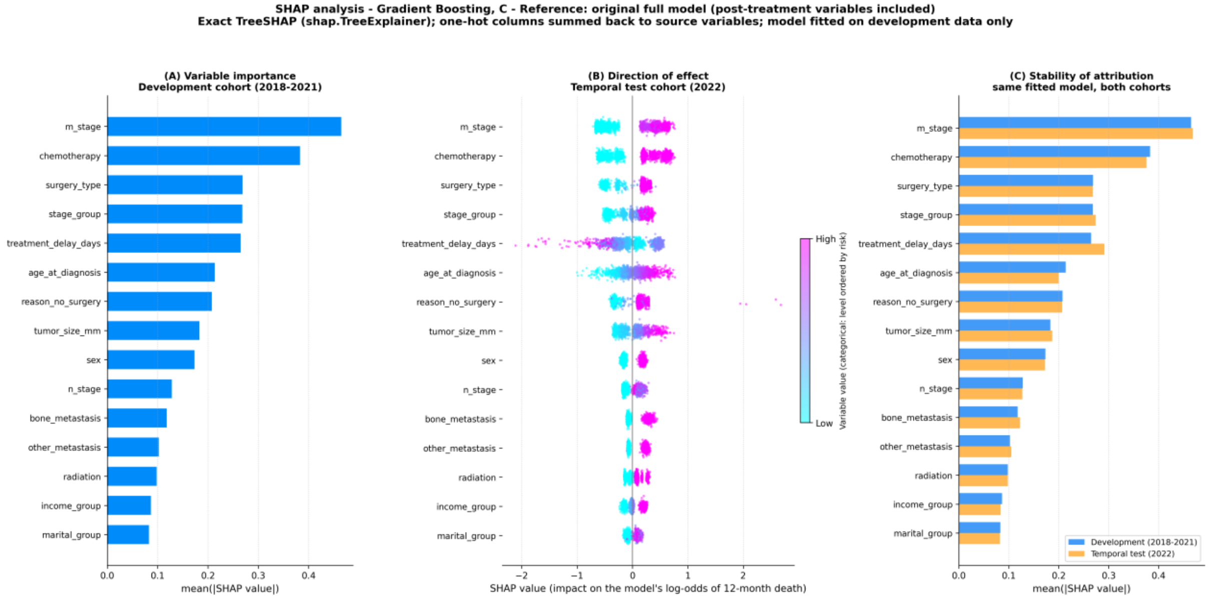

Supplementary Figure S7. Shapley additive explanations for the reference specification (gradient boosting, Specification C). Chemotherapy ranks second overall, and surgery type, treatment delay, reason for no surgery and radiotherapy together account for a large share of the model's explanation, displacing baseline prognostic factors down the ranking. The apparent protective associations of chemotherapy and of definitive surgery reflect confounding by indication, because fitter patients with a better prognosis are those who receive and complete treatment; these attributions must not be read as causal effects of treatment on survival. These values are reported as a reference analysis and are not attainable at initial presentation.

(A) Specification B - diagnosis-only, T/N/M components

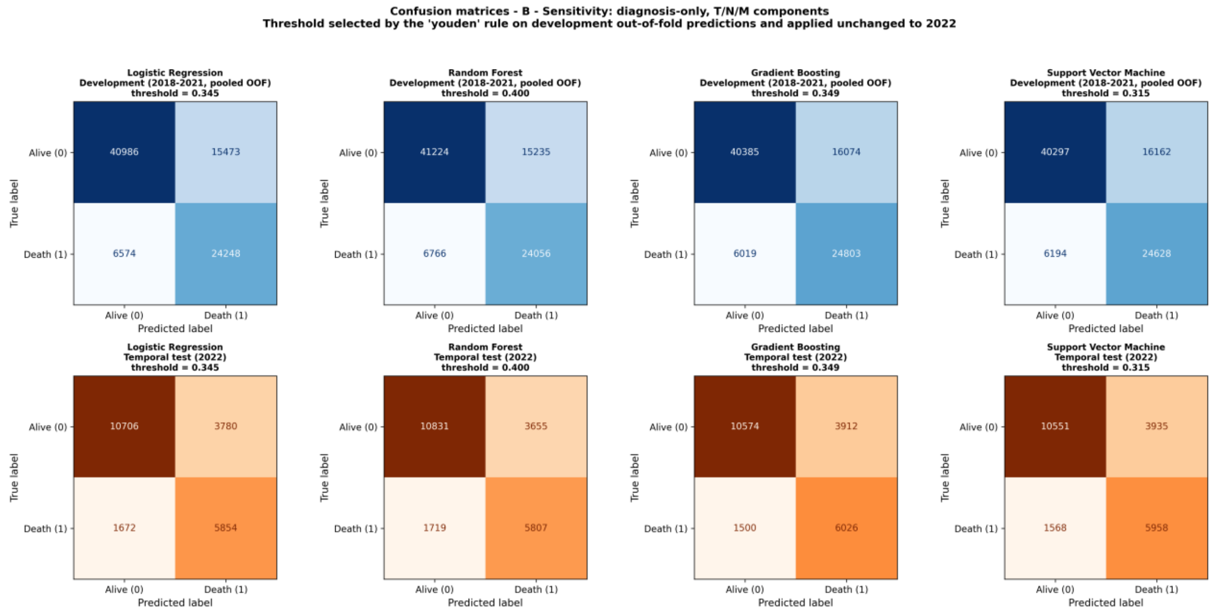

(B) Specification C - post-diagnosis variables included

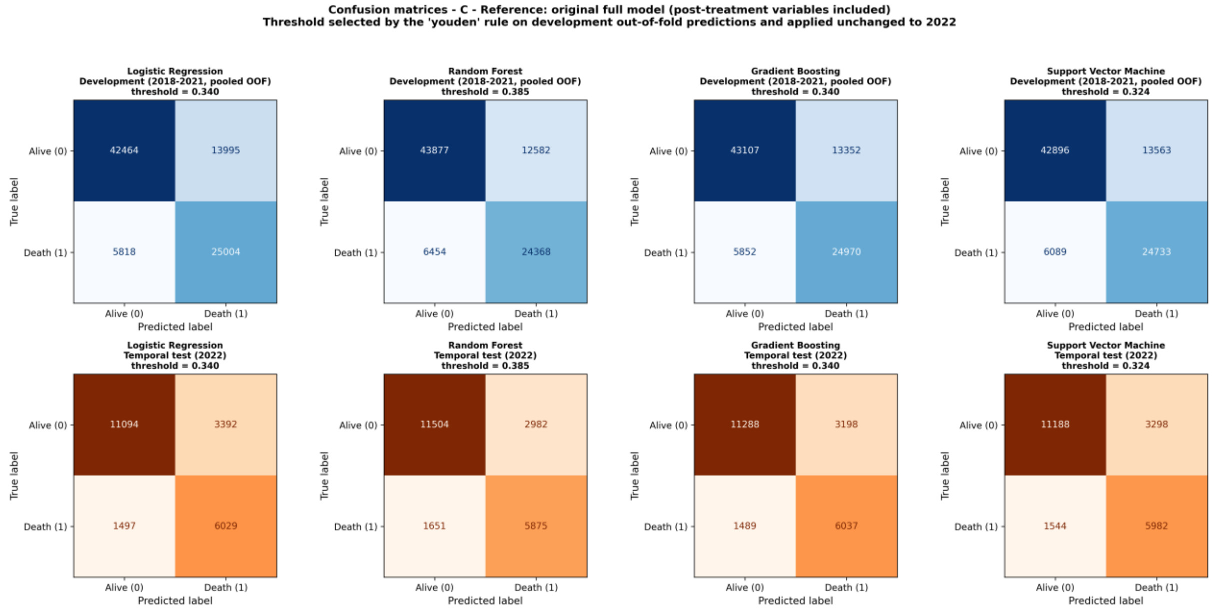

Supplementary Figure S8. Confusion matrices for Specifications B and C at their locked thresholds. In each panel, the upper row is the development cohort and the lower row is the 2022 temporal validation cohort. The threshold shown above each matrix was selected by the Youden rule applied to the pooled development out-of-fold predictions of that specification, then locked and applied unchanged to the 2022 cohort; no threshold optimisation was performed on the validation data. Cells are ordered as true negatives and false positives (upper row of each matrix) and false negatives and true positives (lower row). The higher specificity and positive predictive value under Specification C reflect post-diagnosis information rather than better prognostication at diagnosis.
